# Comprehensive genomic and digital pathology profiling of tobacco‐chewer female oral cancer patients simultaneously with integration of single‐cell datasets identifies clinically actionable patient subgroups

**DOI:** 10.1002/ctm2.70386

**Published:** 2025-07-07

**Authors:** Arnab Ghosh, Siddharth Singh, Tuneer R. Mallick, Shouvik Chakravarty, Supriya Varsha Bhagat, Chitrarpita Das, Kodaganur S. Gopinath, Azeem Mohiyuddin, Arindam Maitra, Partha P. Majumder, Tapas K. Kundu, Nidhan K. Biswas

**Affiliations:** ^1^ Biotechnology Research and Innovation Council‐National Institute of Biomedical Genomics (BRIC‐NIBMG) Kalyani India; ^2^ Biotechnology Research and Innovation Council‐Regional Centre for Biotechnology (BRIC‐RCB) Faridabad India; ^3^ Molecular Biology and Genetics Unit Jawaharlal Nehru Centre for Advanced Scientific Research Bangalore India; ^4^ Laboratory of Signaling and Gene Regulation, Cecil H. and Ida Green Center for Reproductive Biology Sciences University of Texas Southwestern Medical Center Dallas USA; ^5^ Sri Devaraj Urs Academy of Higher Education and Research (SDUAHER) Kolar India; ^6^ John C. Martin Centre for Liver Research and Innovations Kolkata India; ^7^ Indian Statistical Institute Kolkata India

1

Dear Editor,

Genomic alteration landscape of oral squamous cell carcinoma (OSCC) among female patients remains understudied,^1^, ^2^ although certain regions – southeast Asia, including India – show high burden of OSCC among females.^3^ Through generation of whole exome sequencing (WES) and copy‐number‐array from paired tumour and paired normal tissues, and integrative analysis with digital pathology image from same tumour sections, this study identified somatic alterations associated with differential immune infiltration profiles of these tumours (Figure S1). Further integration of large‐scale single‐cell transcriptomics data reveals novel epithelial and immune interactions for this immune suppression which led to identification of two therapeutically actionable subgroups within these patients.

WES analysis from 38 female OSCC patients (Table S1) identified 5141 somatic mutations (of which 3846 were non‐synonymous) with a median of 105.5 mutations per patients with a range of 8–564 (Figure 1A and Table S2). Ten genes, *CASP8* (mutated in 57.89% patients), *TP53* (55.26%), *FAT1* (42.11%), *NOTCH1* (31.58%), *CDKN2A* (23.68%), *HLA‐B* (21.05%), *HRAS* (21.05%), *EPHA2* (15.79%), *PIK3CA* (15.79%) and *ARID2* (13.16%), were found to be significantly mutated (*q *< .1) (Table 1 and Figures 1B and S2). We also detected somatic mutations in *KMT2B*, *HLA‐A*, *PTEN* and *FBXW7* genes which are previously known to be associated with head and neck cancer. Based on somatic mutational landscape of the driver genes, patients could be stratified into four broad groups (Figure 1B). We showed *CASP8* mutations alter pro‐caspase‐8 and active caspase‐8 levels in tumour lysates as evident from western blot assays (Figure S3). We did not find any significant (*p *= .345) differences in frequency of *TP53* somatic mutations in these female patients (55.26%) as compared with male patients in ICGC‐India OSCC cohort (60.92%),^2^ but the frequency was significantly (*p *= .0001) lower than the HPV(−) male patients in TCGA‐HNSC cohort (84.93%) (Table 1). *TP53* mutated patients had significantly (*p *= .035) lower recurrence‐free survival (Figure S4). In contrast, prevalence of *CASP8*, *NOTCH1*, *CDKN2A*, *HRAS*, *HLA‐B* and *EPHA2* mutations were significantly higher (*p *< .026) in these female patients than male patients in ICGC‐India OSCC cohort^1^, ^2^ (Table 1). Oncogenic hotspot somatic mutations in *TP53*, *PIK3CA* or *HRAS* genes were identified in 28.95% of the patients (Figure 1B). These oncogenic somatic mutations occurred significantly (*p *= .0331) more frequently in tumours with *CASP8* mutations (45.45%) than among the remaining (12.5%). The somatic mutations in this cohort were found to be contributed by 12 frequently detected (≥10% tumours) somatic‐mutational‐signatures linked to tumour aging, smoking, APOBEC hyperactivity, UV and defective DNA repair (Figure 1C).

**FIGURE 1:**
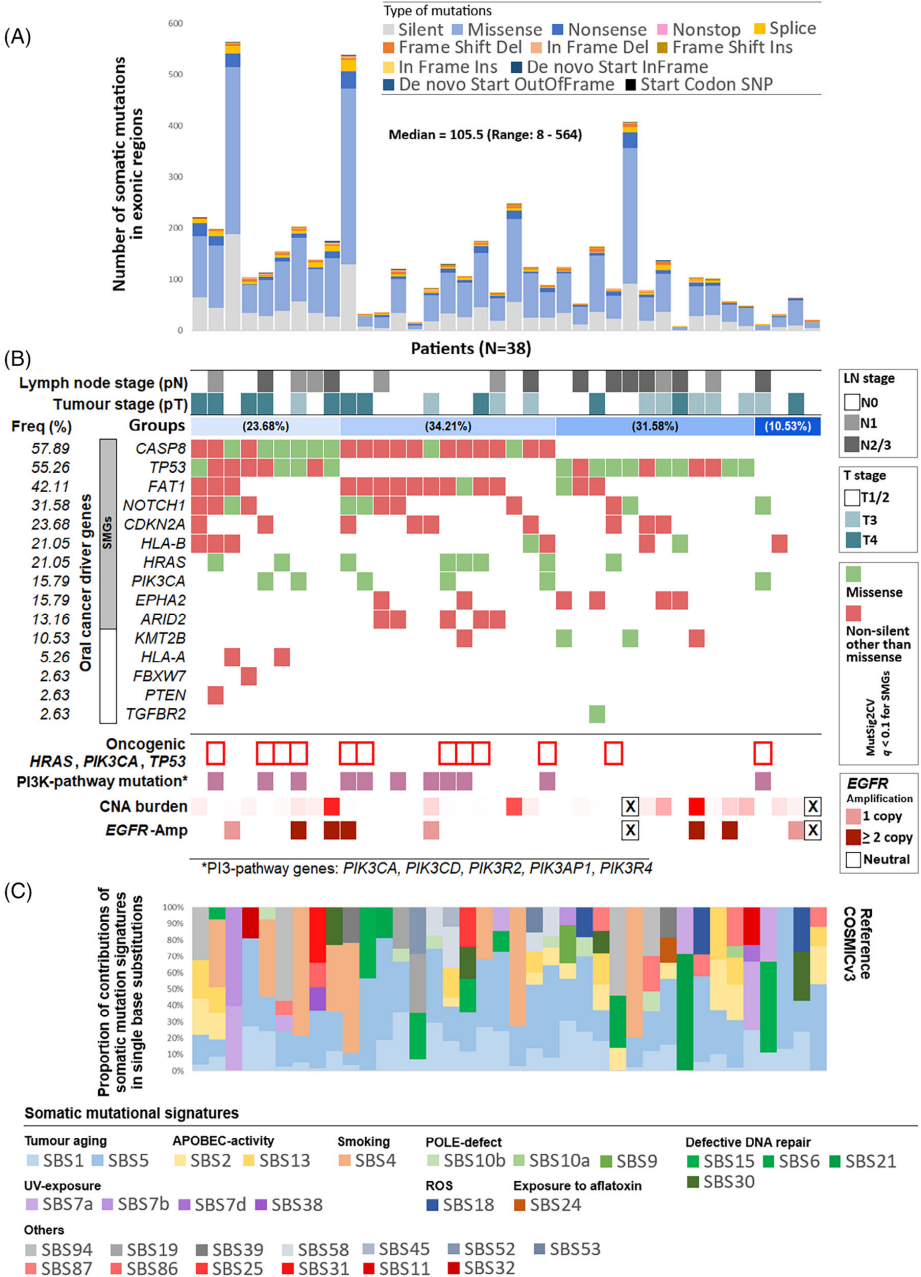
Somatic mutational landscape of oral squamous cell carcinoma of female patients from a southern Indian cohort. (A) Numbers and distributions of different types of somatic mutations in 38 patients. We have detected a median of 105.5 somatic mutations including both silent and non‐silent in these oral tumours. (B) Somatic mutational (*N* = 38) and copy number alteration status (*N* = 36 patients for whom genotype CNV array data passed quality control and analysed) for oral cancer driver genes. We found 10 genes to be significantly (*q *< .1, MutSig2CV) mutated (highlighted) along with mutations in other known oral and head and neck cancer driver genes. Broadly, four broad molecular sub‐groups were identified – patients with (1) both *CASP8* and *TP53* mutations, (2) *CASP8* mutation without *TP53* mutation, (3) *TP53* mutation without *CASP8* mutation and (4) Both *CASP8* and *TP53* wild‐type. We detected oncogenic mutations (in *TP53*/*HRAS*/*PIK3CA*) in 22.92% patients and *EGFR* amplification in 17.4% patients. (C) Relative contributions of various mutagenesis processes pertaining to heterogeneous somatic mutational signatures in female oral tumours. 31 COSMICv3 mutational signatures were detected with predominant signatures like SBS1 and SBS5 linked to tumour aging, SBS2 and SBS13 linked to APOBEC enzymatic activity, SBS4 linked to tobacco smoking and so on. Multiple mutational signatures related to defective DNA repair and UV exposure was detected in these tumours. One patient harboured SBS24 which is linked to exposure to aflatoxin.

**TABLE 1:** Comparison of frequency of somatic mutations of SMGs with ICGC – oral cancer cohort and TCGA‐HNSC cohort.

SMG			dbGENVOC	*p* value		*p* value
Rank	Gene	Female centric OSCC cohort	ICGC‐India	(Fisher's exact test, one‐sided)	TCGA HNSCC	(Fisher's exact test, one‐sided)
		Female (*N* = 38)	Male (*N* = 87)		HPV(−) Male (*N* = 292)	
1	*TP53*	21 (55.26%)	53 (60.92%)	.3454	248 (84.93%)	.0001
2	***CDKN2A***	9 (23.68%)	5 (5.75%)	.0059	73 (25.00%)	.5196
3	***HLA‐B***	8 (21.05%)	6 (6.90%)	.026	11 (3.77%)	.0004
4	***CASP8***	22 (57.89%)	33 (37.93%)	.0307	28 (9.59%)	.0001
5	***HRAS***	8 (21.05%)	7 (8.05%)	.0429	17 (5.82%)	.0037
6	***EPHA2***	6 (15.79%)	2 (2.30%)	.0098	13 (4.45%)	.0138
7	*FAT1*	16 (42.11%)	23 (26.44%)	.0644	65 (22.26%)	.0087
8	*ARID2*	5 (13.16%)	10 (11.49%)	.502	9 (3.08%)	.0143
9	***NOTCH1***	12 (31.58%)	9 (10.34%)	.0049	50 (17.12%)	.0319
10	*PIK3CA*	6 (15.79%)	12 (13.79%)	.4832	40 (13.70%)	.4414

*Note*: 6 significantly mutated genes (SMG) showing statistically significant difference in mutation frequency in the female OSCC cohort as compared to male OSCC cohort in India are highlighted.

The median copy‐number‐alteration burden was 0.09% ranging between 0.0016 and 23.5%, with significant (*q *< .1) amplification of 3p, 8p, 8q, 19p and 19q, and deletion of 3q, 8q, 9p, 9q and 14q chromosome arms (Table S3). Significant focal amplification of *ALDH1L1* (56.5%) and *EGFR* (17.4%) (Figure 1B) were identified. Alongside, focal amplification of *KCNJ11*, and deletions of *CDK7*, *CDKN2A*, *BRAF* and *FAT1* genes were identified.

Somatic alteration patterns are often shown to influence immune infiltration in solid tumours. In this study, we have utilised whole slide imaging (WSI) data generated (20×‐magnification) from haematoxylin–eosin‐stained (H&E) tumour tissue sections for a subset of patients (Figure 2A) to infer immune infiltration. Recently developed deep‐learning model^4^ was utilised to quantify tumour infiltrating leukocytes (TILs) (Table S4 and Figure S5) from WSIs, followed by validation using an orthogonal model (Figure S6) at cell level (Figure 2B). From these WSIs, a median TILs infiltration of 4.84% with a range between 0 and 27.19% was detected for these tumours. Based on the distribution of the proportion of TILs, the patients could be stratified in two groups – (a) low‐TIL (with 0–0.5% TIL) and (b) high‐TIL (with 4.84–27.19% TIL) (Table S4). The somatic mutational burden in these tumours were not correlated with the proportion of TILs. We undertook an unbiased approach to correlate somatic alteration status (mutation or copy number alterations) with TIL infiltration and found – tumours with low‐TIL are significantly (*p *= .007) more enriched with *EGFR* copy number amplification (71.4%) as compared with tumours with high‐TIL (0%). The average fraction of TILs in *EGFR*‐amplified tumours was 0.17% ranging between 0 and 0.5%, while the remaining tumours had an average of 12.5% TILs ranging between 0.14 and 27.19% (Figure  and Table S4). In contrast, *CASP8*‐mutated tumours (enriched for co‐occurrence of other oncogenic‐mutations) showed significantly (*p *= .026) higher infiltration of TILs (median = 9.98%), than the rest (0.17%) (Figure 2B and Table S4). These findings were validated in TCGA‐HNSC cohort (Figure S7A).

**FIGURE 2:**
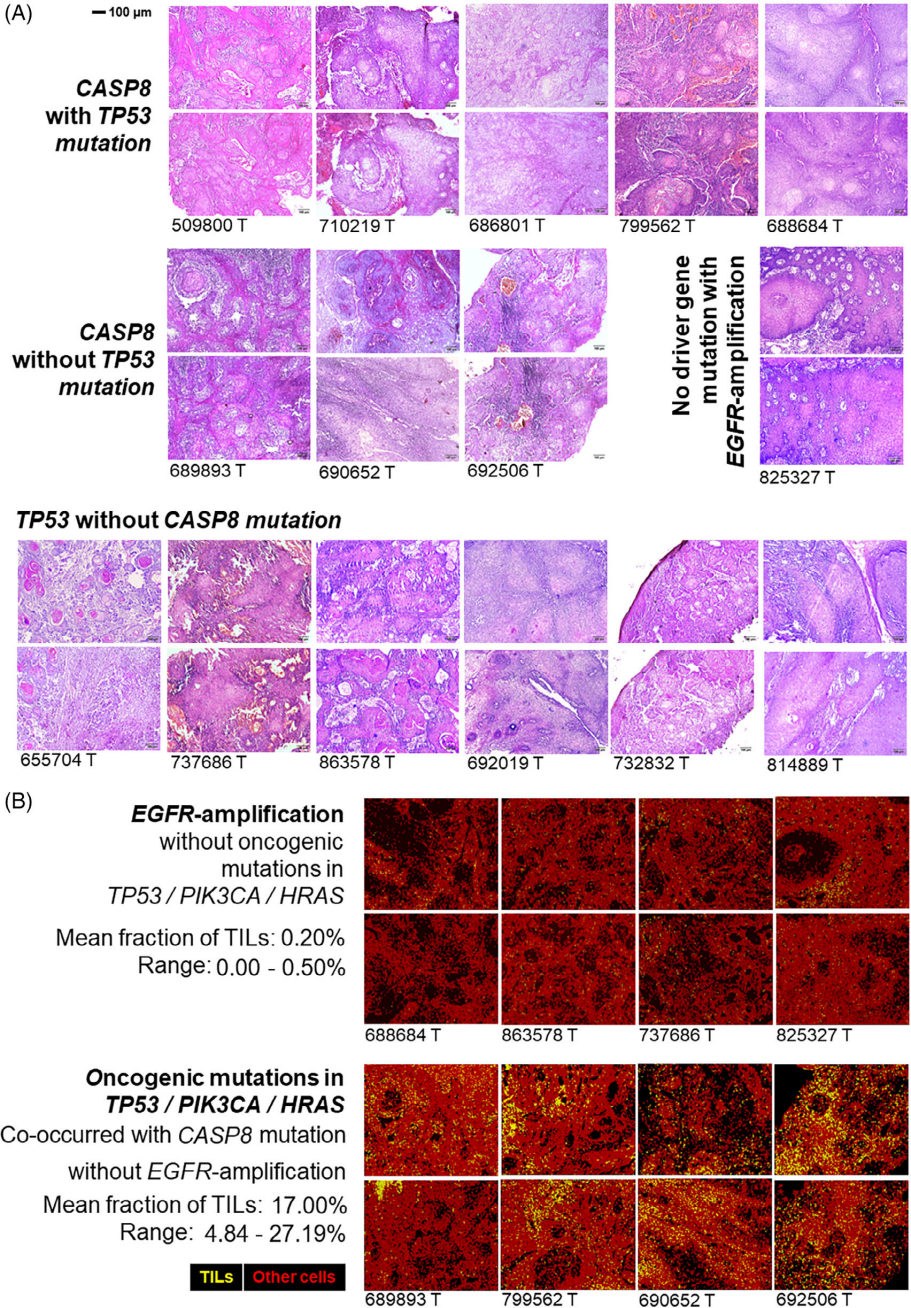
(A) H&E‐stained images from tumour core tissue section (two different field for each patient) for a subset of 15 patients – representative of four different sub‐groups (described in Figure 1): – patients with (1) both *CASP8* and *TP53* mutations (*n* = 5), (2) *CASP8* mutation without *TP53* mutation (*n* = 3), (3) *TP53* mutation without *CASP8* mutation (*n* = 6) and (4) Both *CASP8* and *TP53* wild‐type (*n* = 1) (this patient had *EGFR* amplification as driving event). The histopathological features of the patient with only *EGFR* alteration as driving event was found to be distinct from the remaining tumours. We have quantified the extent of tumour infiltrating lymphocytes (TILs) in each of the 15 tumours by digital pathology which showed – (1) tumours with *EGFR* amplification are depleted with TILs and in contrast (2) tumours with oncogenic somatic mutations (in *TP53*/*PIK3CA*/*HRAS* genes) (also harbour co‐occurring *CASP8* mutations) but without *EGFR* amplification harboured high immune infiltration (TIL).

As *EGFR* expression was positively correlated with its genomic‐copy‐number in TCGA‐HNSC cohort (Figure S7B), to further elucidate the plausible mechanism of EGFR driven immune suppression, we have created and analysed an integrated gene expression atlas of 66,809 single cells from 55 oral cavity tumours (Figure 3A–E). We show that tumour epithelial cells with high EGFR expression featured – (a) significant down‐regulation of (a.1) antigen‐processing and presentation (*HLA‐A*, *HLA‐B*, *HLA‐DRA*, *B2M*, etc.), (a.2) immune‐stimulating genes (*CXCL17*, *LCN2* and *SAA1*), and (b) significant up‐regulation of (b.1) pro‐angiogenic factors *VEGFB* and *FGFBP1*, (b.2) glutathione biosynthesis related gene *GPX2*, and its interacting partners *AKR1C1*, *AKR1C2* and *AKR1C3* (Figure 3F). High EGFR expressing tumour cells were shown to have elevated epithelial‐to‐immune interactions through (1) PTN (interacting with NCL on immune cells), (2) PLAU (interacting with PLAUR on macrophages), (3) ADM (interacting with CALCRL on pDCs) and (4) MDK (interacting with ITGA4 and ITGB1 on macrophages) (Figure 3G,H) – potentially facilitating pro‐tumourigenic microenvironment. Whereas interactions necessary of T‐cell activation, like MIF (interacting with CXCR4, CD74 and CD44), PPIA (interacting with BSG) and CXCL16 (interacting with CXCR6 on CD8+ T‐cells) were down‐regulated in high EGFR expressing tumour cells. Moreover, we show that these EGFR‐high tumour cells overexpressed *LGALS3*, *CD59*, laminins (*LAMB1* and *LAMB3*), *PROS1* and so on that induced *LAG3* and *CD44* expression in the associated CD8+ T cells (Figure 3I–K) featuring an exhaustion state. Recently *EGFR*‐amplification was shown to decrease immune‐infiltration in lung^5^ and gastroesophageal‐adenocarcinoma.^6^ Detailed methodology and results are available as Supporting Information.

**FIGURE 3:**
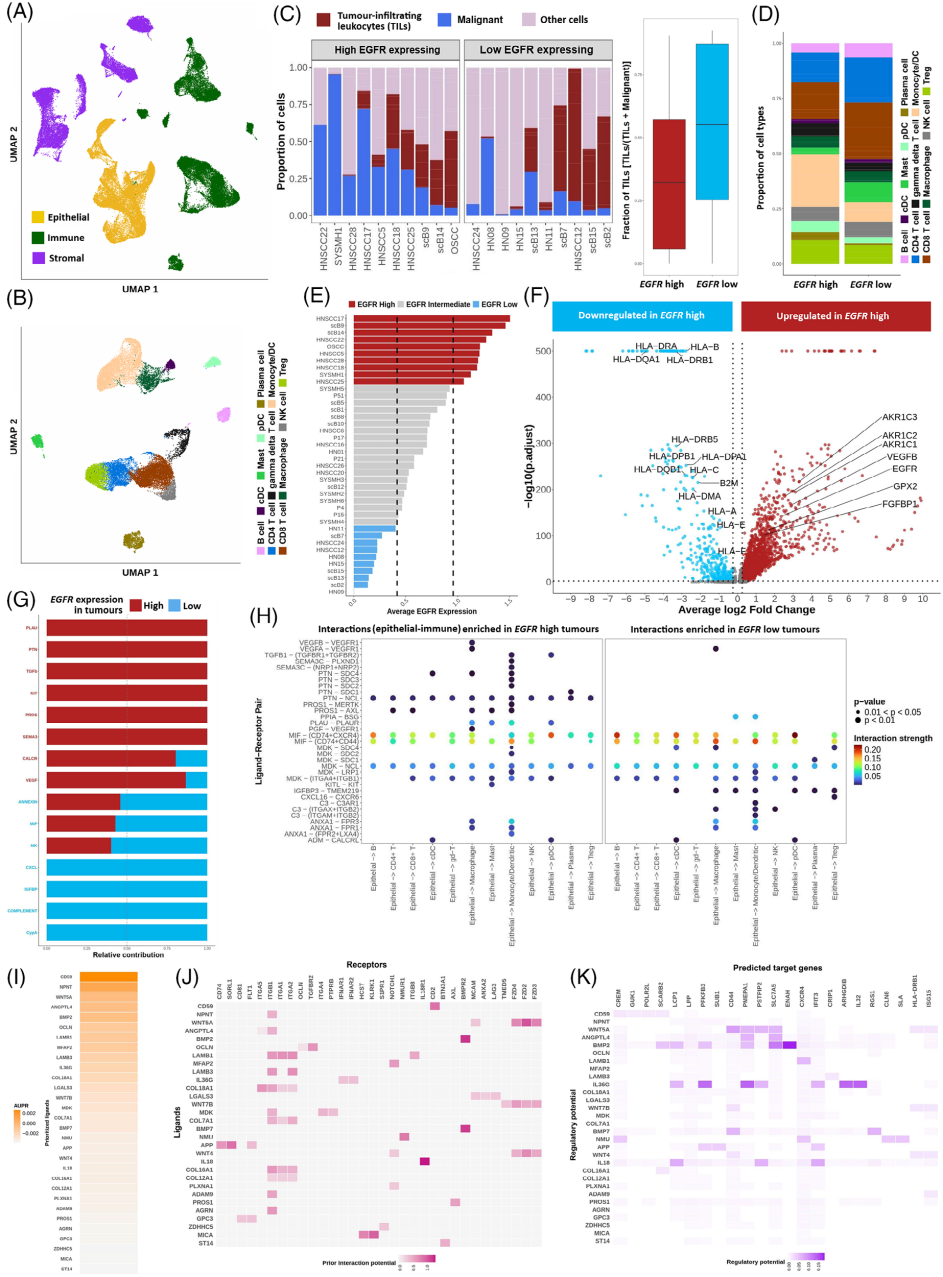
(A) UMAP showing three major cell type lineages in the integrated dataset. (B) UMAP showing 12 immune cell types identified. (C) Bar‐plot showing proportion of TILs (brown) in the TME of *EGFR*‐high (left) and *EGFR*‐low (right) tumours. Proportion of TILs compared with malignant cells in EGFR‐high (red) versus EGFR‐low (blue) tumours. (D) Proportion of different immune cell types in *EGFR*‐high versus Low tumours. (E) Classification of tumours into *EGFR*‐high (red) and *EGFR*‐low groups (blue) based on average *EGFR* expression in epithelial cells. (F) Genes up‐regulated in *EGFR*‐high versus *EGFR*‐low tumours. (G) Relative contribution of significantly enriched outgoing signalling pathways in *EGFR*‐high (red) and *EGFR*‐low (blue) epithelial cells. (H) Interactions between ligands expressed by epithelial cells and receptors expressed by immune cell types in *EGFR*‐high and *EGFR*‐low tumours. Left panel shows interactions that are enriched in *EGFR*‐high tumours, while the right panel shows interactions that are enriched in *EGFR*‐low tumours. (I) Top 30 ligands expressed by epithelial cells of *EGFR*‐high tumours inferred by NicheNet. (J) Interaction potential between ligands expressed by epithelial cells and receptors expressed by CD8+ T cells in *EGFR*‐high tumours. (K) Regulatory potential of inferred ligands on target genes in CD8+ T cells of EGFR‐high tumours.

This study provides novel insights into *EGFR*‐amplification mediated immune suppression in oral tumours, which was present in 22.22% of tumours from female OSCC patients. We identified key epithelial–immune interactions in high *EGFR* expressing tumours such as LGALS3–LAG3, PLAU–PLAUR, MDK–(ITGA4+ITGB1) which can be therapeutically targeted with available inhibitors such as relatlimumab,^7^ upamostat^8^ and natalizumab^9^ alongside currently prescribed anti‐EGFR cetuximab^10^ to increase immune infiltration within tumour for potential prognostic benefit. Additionally, the results suggest tumours with oncogenic somatic mutations (found in 31.58% female OSCC patients) that showed comparatively high immune infiltration may benefit from a combination alpelisib or tipifarnib (targeting *PIK3CA* or *HRAS*)^11^ and adjuvant immune‐checkpoint inhibitors.

## AUTHOR CONTRIBUTIONS

N. K. B., T. K. K. and P. P. M. conceptualised the project. T. K. K., S. S., K. S. G. and A. Z. M. coordinated patient recruitment, biospecimen collection. A. M. coordinated sequence data generation through the Indian national genomics core. A. G., S. C., C. D. and N. K. B. performed NGS data analysis. T. R. M., A. G. and N. K. B. integrated publicly available scRNAseq data and interpreted. S. S., S. V. B. and T. K. K. generated histopathology and western blot data. A. G. and N. K. B. integrated genomic and digital pathology data. A. G., S. S., T. R. M., S. C., C. D., P. P. M., T. K. K. and N. K. B. wrote the manuscript.

## CONFLICT OF INTEREST STATEMENT

The authors declare no conflict of interest.

## FUNDING INFORMATION

This study was funded by Department of Biotechnology, Government of India via the Virtual National Oral Cancer Institute (VNOCI) Grant No. BT/PR17576/MED/30/1690/2016.

## ETHICS STATEMENT

This study was approved by the ethics committee of Sri Devaraj Urs Academy of Higher Education and Research (Comprising Sri Devaraj Urs Medical College), Kolar, India [No. SDUAHER/KLR/R&D /81 /2018‐19]. Data and bio‐specimens were collected with voluntary and informed consent.

## Supporting information



Supporting Information

Supporting Information

Supporting Information

Supporting Information

Supporting Information

Supporting Information

Supporting Information

Supporting Information

Supporting Information

Supporting Information

Supporting Information

## Data Availability

Whole exome sequence data have been deposited to Indian Biological Data Center (IBDC) and European Nucleotide Archive (ENA). It can be accessed through study accession – IBDC (INDA‐CA): INCARP000295 and ENA: PRJEB74692.
