# Supplementary material for: Comprehensive genomic and digital pathology profiling of tobacco‐chewer female oral cancer patients simultaneously with integration of single‐cell datasets identifies clinically actionable patient subgroups
Source: Clin Transl Med. 2025 Jul 7;15(7):e70386. doi: 10.1002/ctm2.70386 (PMC12230625; doi:10.1002/ctm2.70386)

Figure S1: Whole exome sequencing (WES) and copy-number array data generation was performed from paired tumour and blood tissue samples from 38 female OSCC-GB patients. The somatic alteration data of these tumours were integrated with H&E-stained digital pathology data to identify genomic features associated with immune cell infiltration within the tumour microenvironment. Further multiple publicly available single-cell data were integrated for validation and generation of additional insights on tumour-immune interactions.

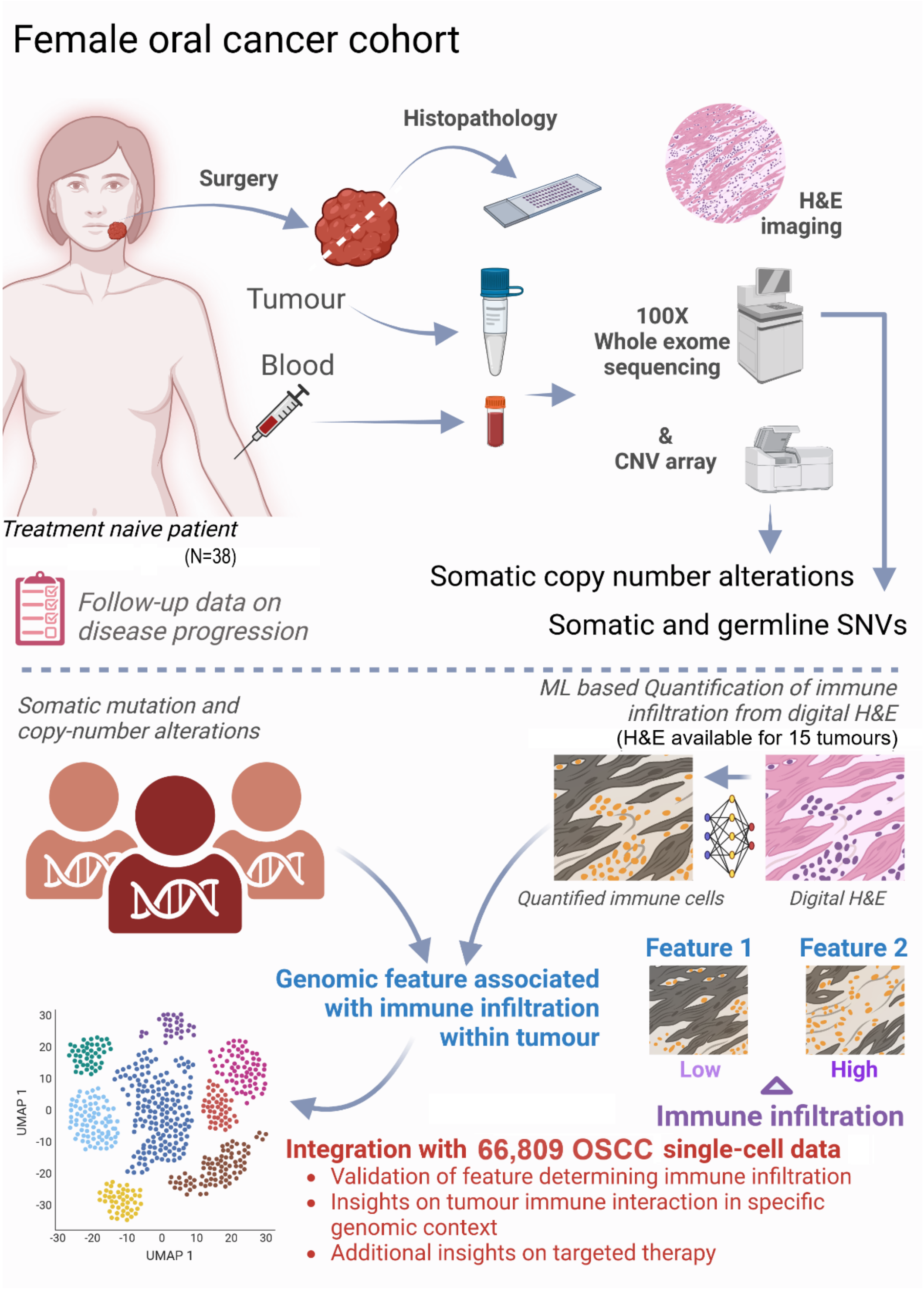

Figure S2: Plot visualizing the distribution of different types of mutations across driver genes within their protein domains. If the mutations are druggable, the corresponding oncoKB annotations were provided for each driver gene.

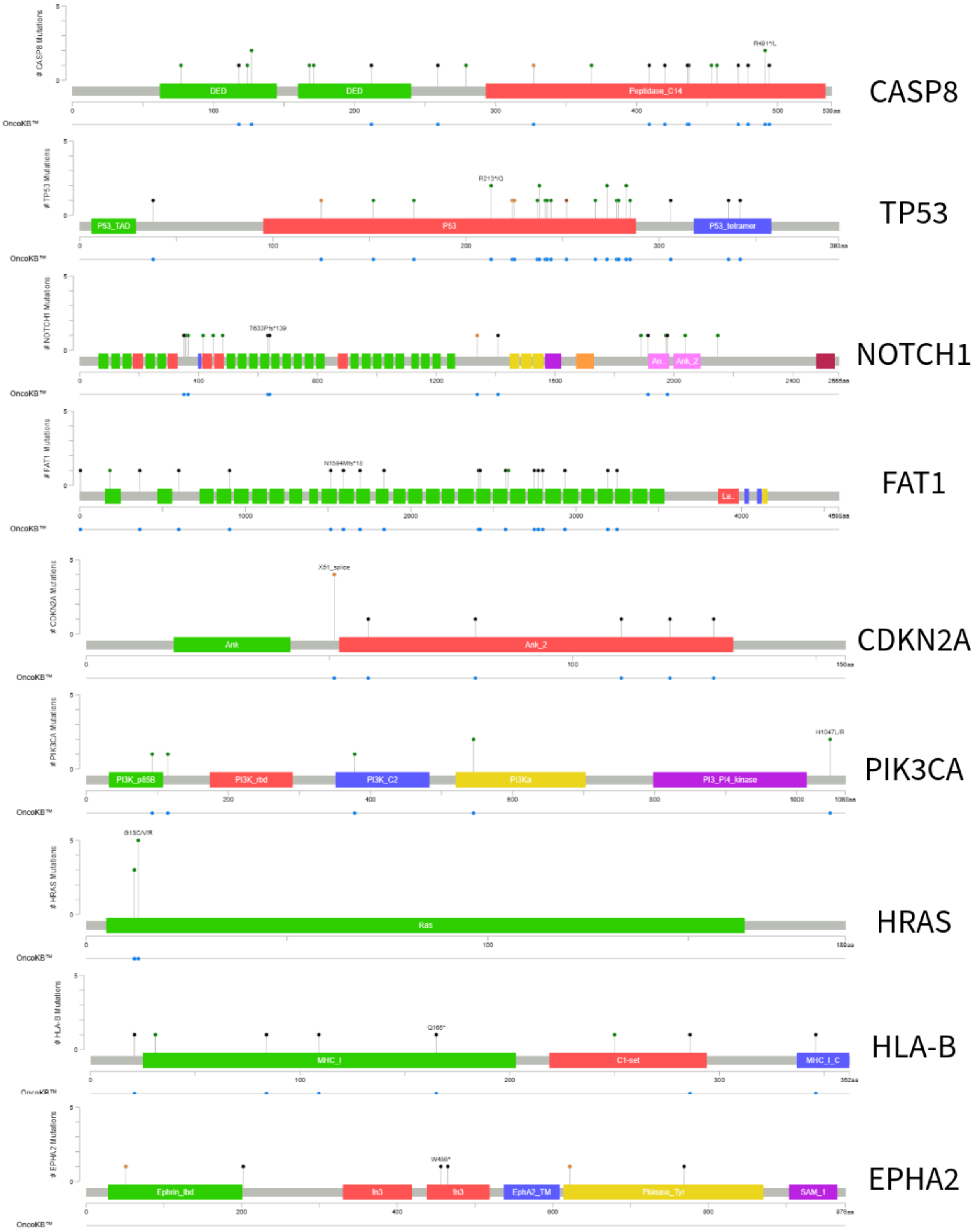

Figure S3: Caspase-8 expression level in 9 patients' tumor samples by western blot analysis. Expression of β-actin used as a loading control. Expression of both procaspase-8 and cleaved caspase-8 were detected in tumor samples with caspase-8 antibody.

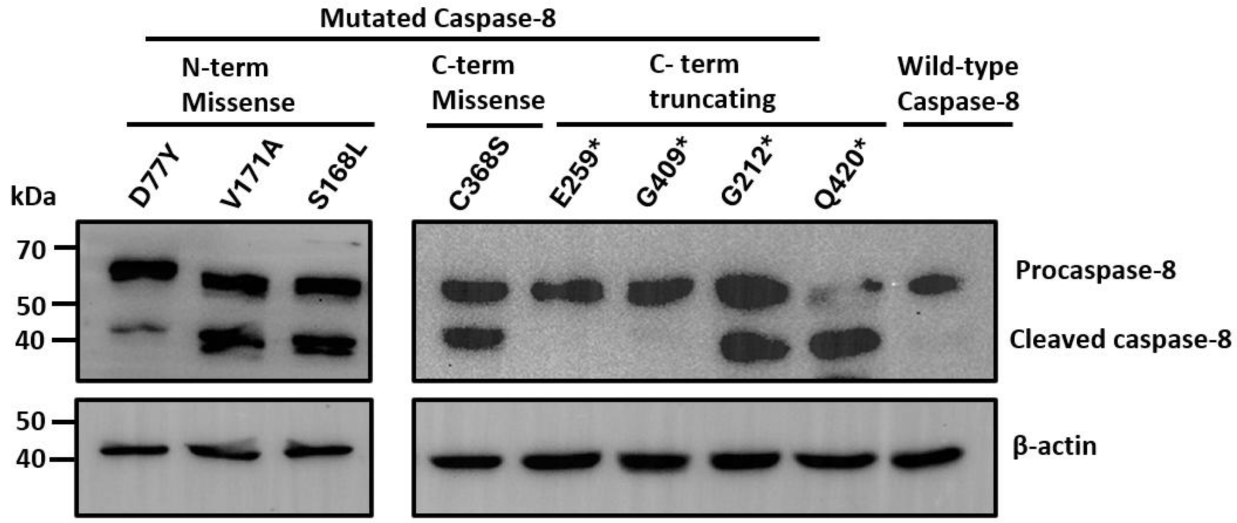

Figure S4: The duration of disease-free survival (DFS) of *TP53* mutated patients were significantly (p = 0.035, Log-rank test) shorter than the remaining patients.

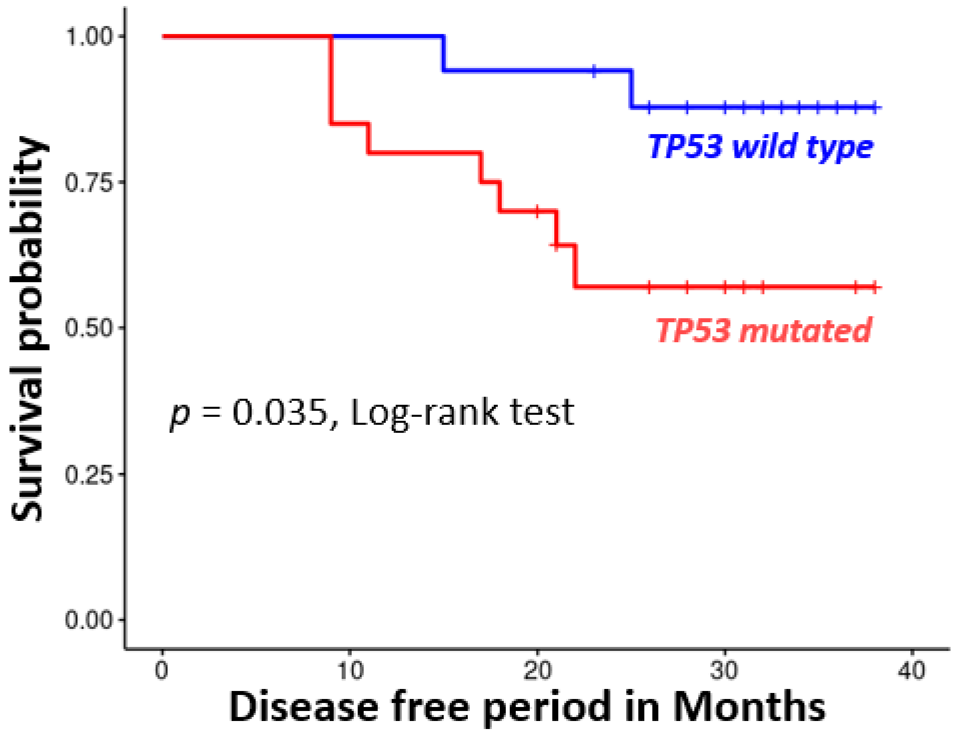

Figure S5: Quantification of TILs based on InceptionResNetV2 model provided with TILScout package (Zhang *et al.*, 2025) from 20X whole slide images (WSI) generated for a subset of tumours. Tumours with *EGFR* copy number amplification showed lower infiltration of TILs (0 – 0.50%). Tumours with oncogenic somatic mutations in *TP53*, *HRAS*, or *PIK3CA*, without *EGFR* amplification (these tumours also had *CASP8* somatic mutations) showed higher abundance of TILs (4.84 – 27.19%). One tumour having both oncogenic somatic mutation, and *EGFR* amplification showed low proportion of TILs (0.07%) indicating EGFR amplification may be a dominant factor in respect to infiltration of TILs.

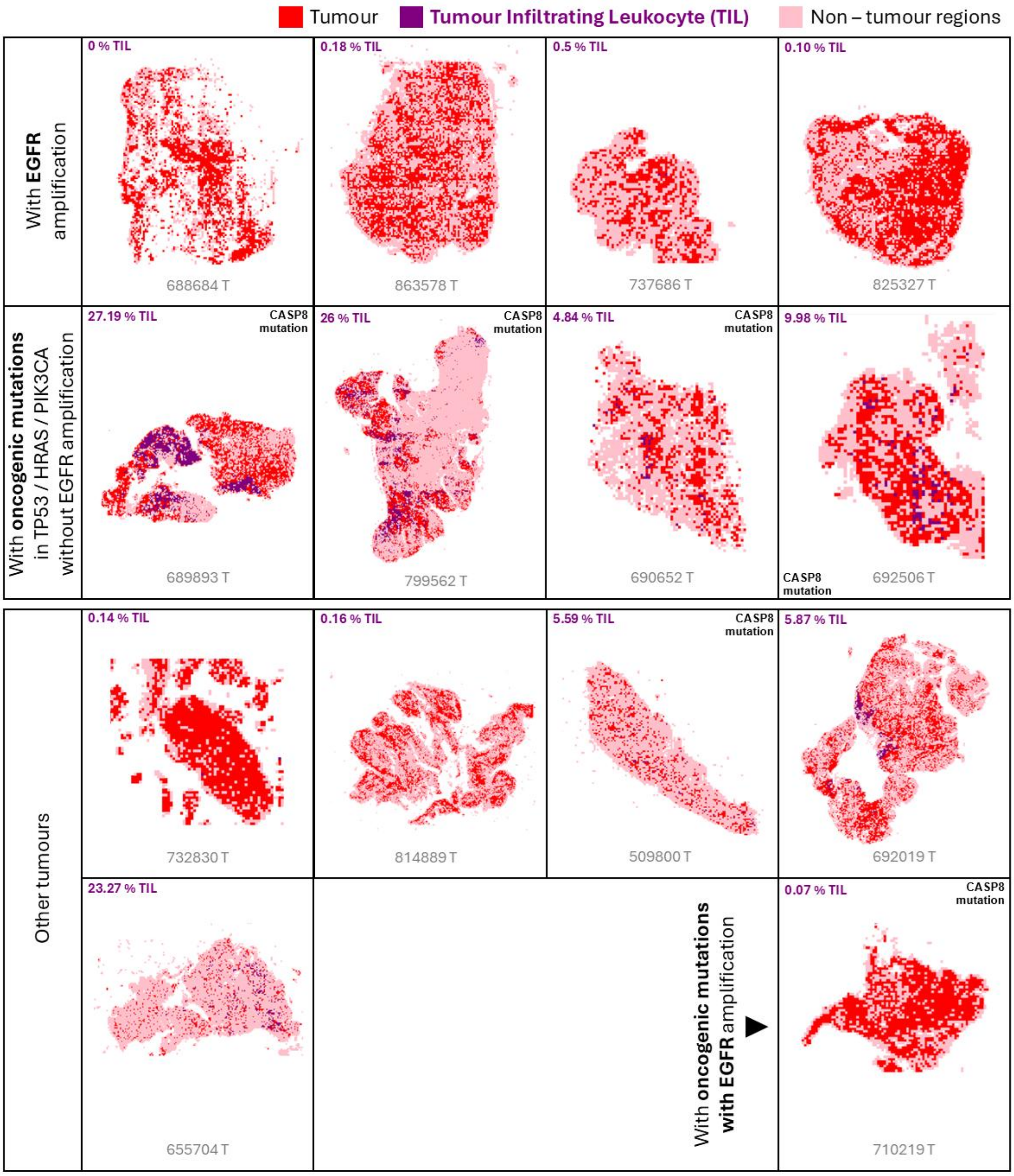

Figure S6: An orthogonal approach for detection of leukocytes at cell level from sub sections of WSI. (A) Manual annotation of segments enriched with TILs (5 samples, yellow) and non-TILs (19 samples with diverse morphology, red) for training of model through object classifier in QuPath software. Representative image of detected TILs (yellow) from tumours with (B) high and (C) low infiltration. (D) Validation of segment level TILs estimated from 20X WSI through TILScout, with cell level prediction through QuPath trained model.

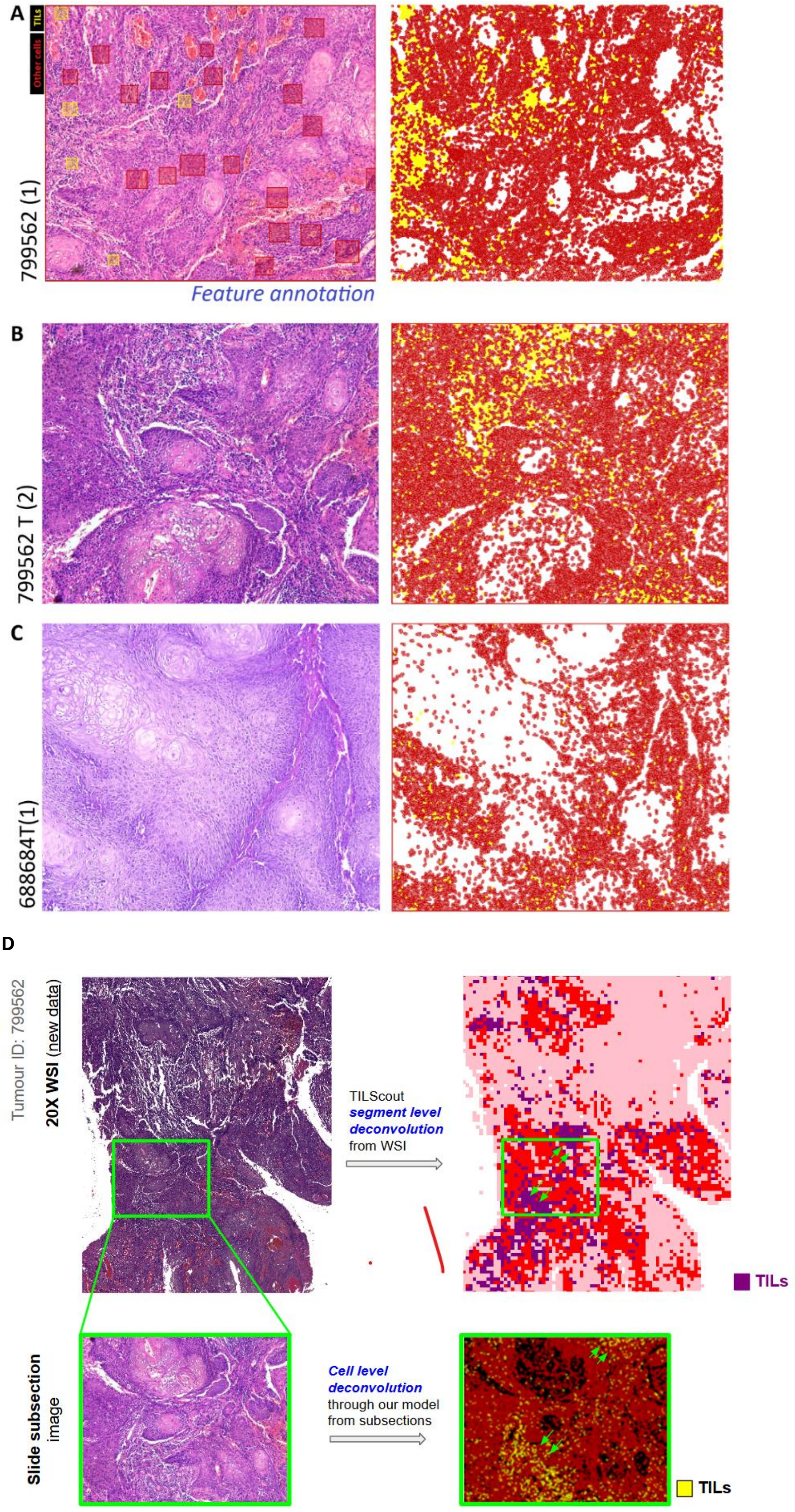

Figure S7: (A) Immune score computed through ESTIMATE algorithm showing negative and positive association with EGFR amplification and presence of oncogenic mutational signature respectively, in TCGA-HNSC tumours. (B) In TCGA-HNSCC cohort, EGFR expression was significantly positively correlated (r=0.5, p < 2.2e-16, Pearson's correlation) with its genomic copy number status [sample size – EGFR shallow deletion: 20, diploid (copy neutral): 261, gain: 155, amplification: 52].

A **B**

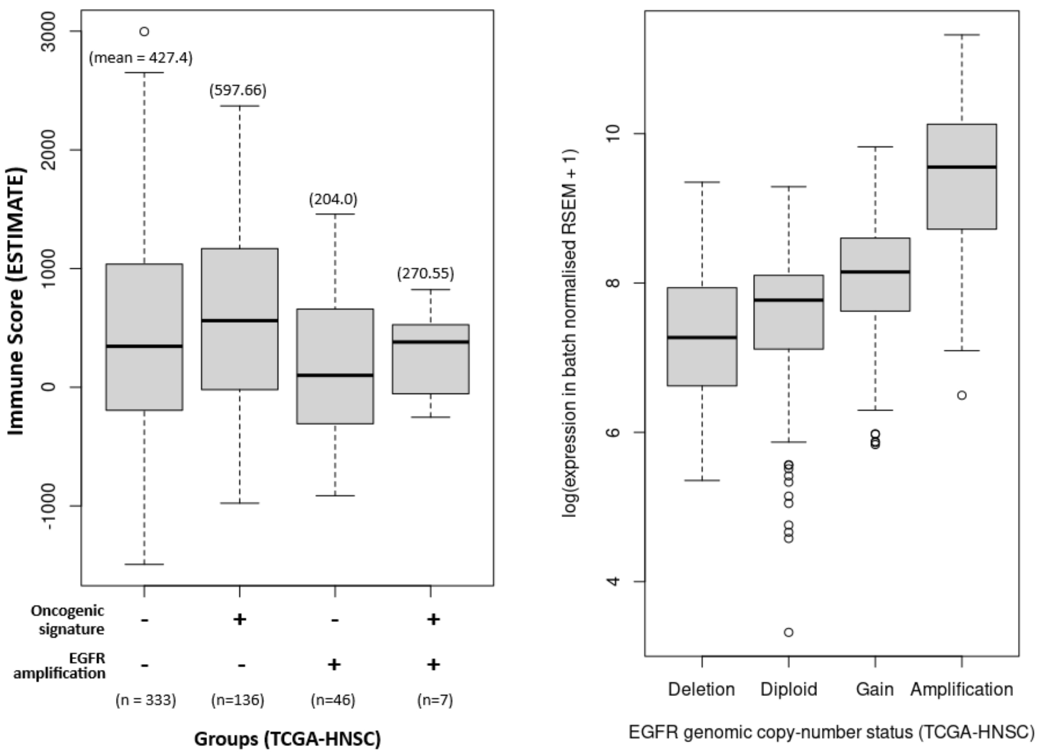

Figure S8: Pathway enrichment analysis by functional interaction (FI) networks (A) including all somatic mutations in these oral tumours and (B) excluding oral cancer driver genes to capture the passenger mutational effect.

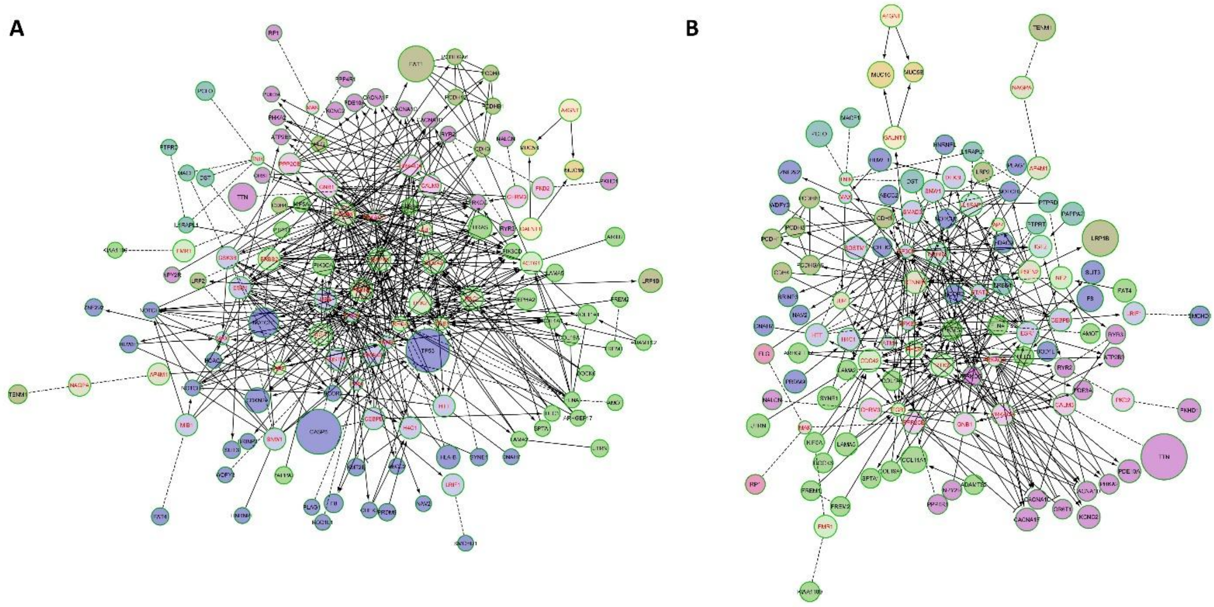

Figure S9: (A) Distribution of local inverse Simpson's index (LISI) values before and after study-specific batch-effect removal in scRNAseq data. (B) Dotplot showing expression of cell-lineage specific markers in the integrated single-cell atlas of head and neck tumors across 6 studies. (C) Dotplot showing expression of 12 immune cell specific markers. (D) Distribution of per-sample average CopyKAT-inferred CNV profiles of EGFR gene in EGFR high (red) and low (blue) expressing tumors. (E-F) Outgoing and incoming signalling strengths of epithelial and immune cell types in EGFR-High (E) and EGFR-Low tumors (F). (G) Heatmap showing differential number of ligand-receptor interactions between epithelial and immune cell types in EGFR-High (red) and EGFR-Low (blue) tumors. (H) Relative contribution of significantly enriched signalling pathways in EGFR-High (red) and EGFR-Low (blue), where epithelial cells are senders and CD8 T cells are receivers.

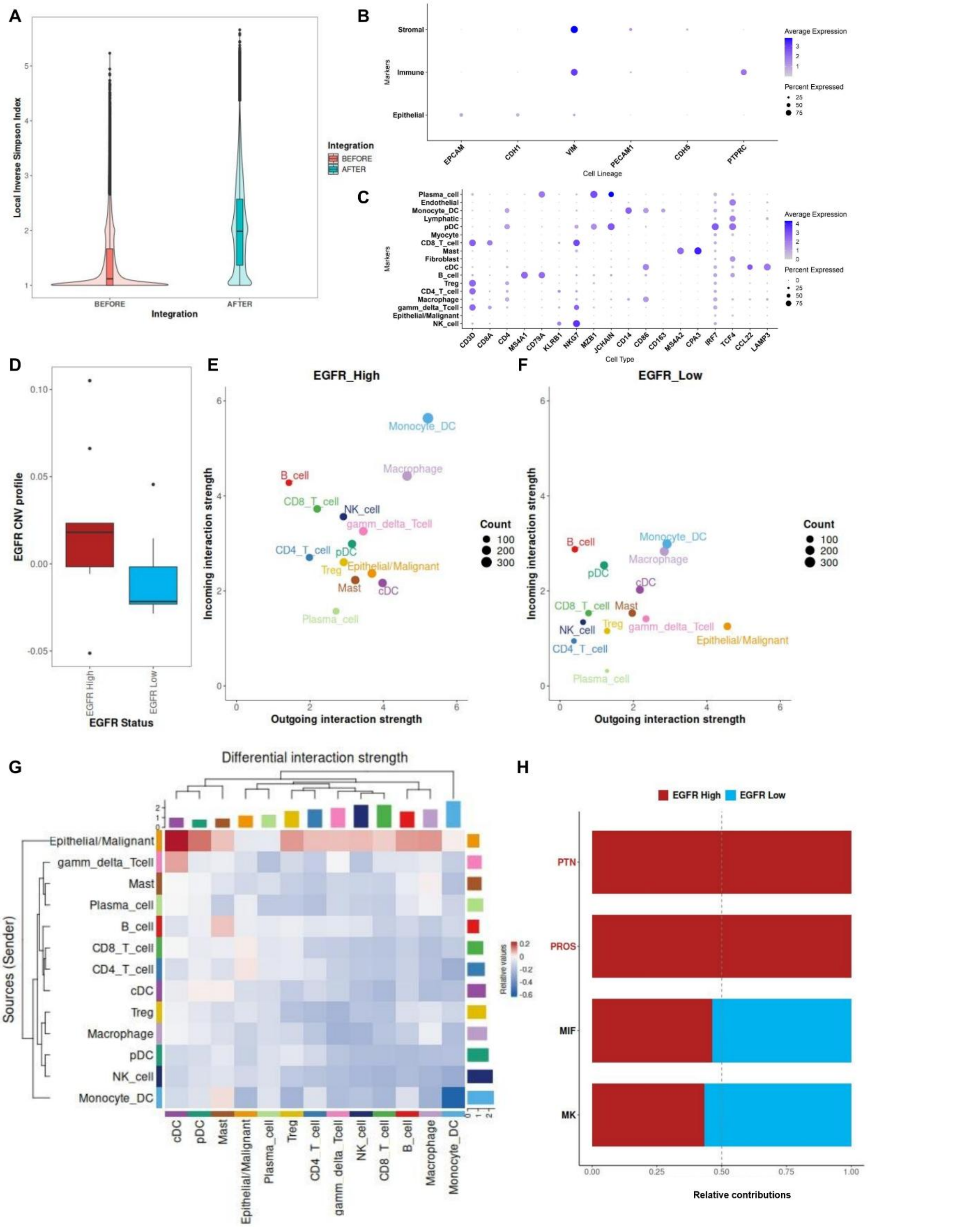

Figure S10: Intra-tumour heterogeneity revealing heterogeneous life-histories of oral tumours. (A) The tumour heterogeneity MATH score did not correlate with tumour stage (pathological), lymph node metastasis status, or number of driver gene (oral cancer specific) mutations in this cohort. (B) Tumours with high MATH score (i.e., higher intra-tumour heterogeneity) are enriched with TP53 mutational status. (C) Distributions of variant allele fractions (surrogate of clonal architecture) of somatic mutations in these patients with driver gene mutational status – revealing both inter- and intra-tumour heterogeneity in oral tumours.

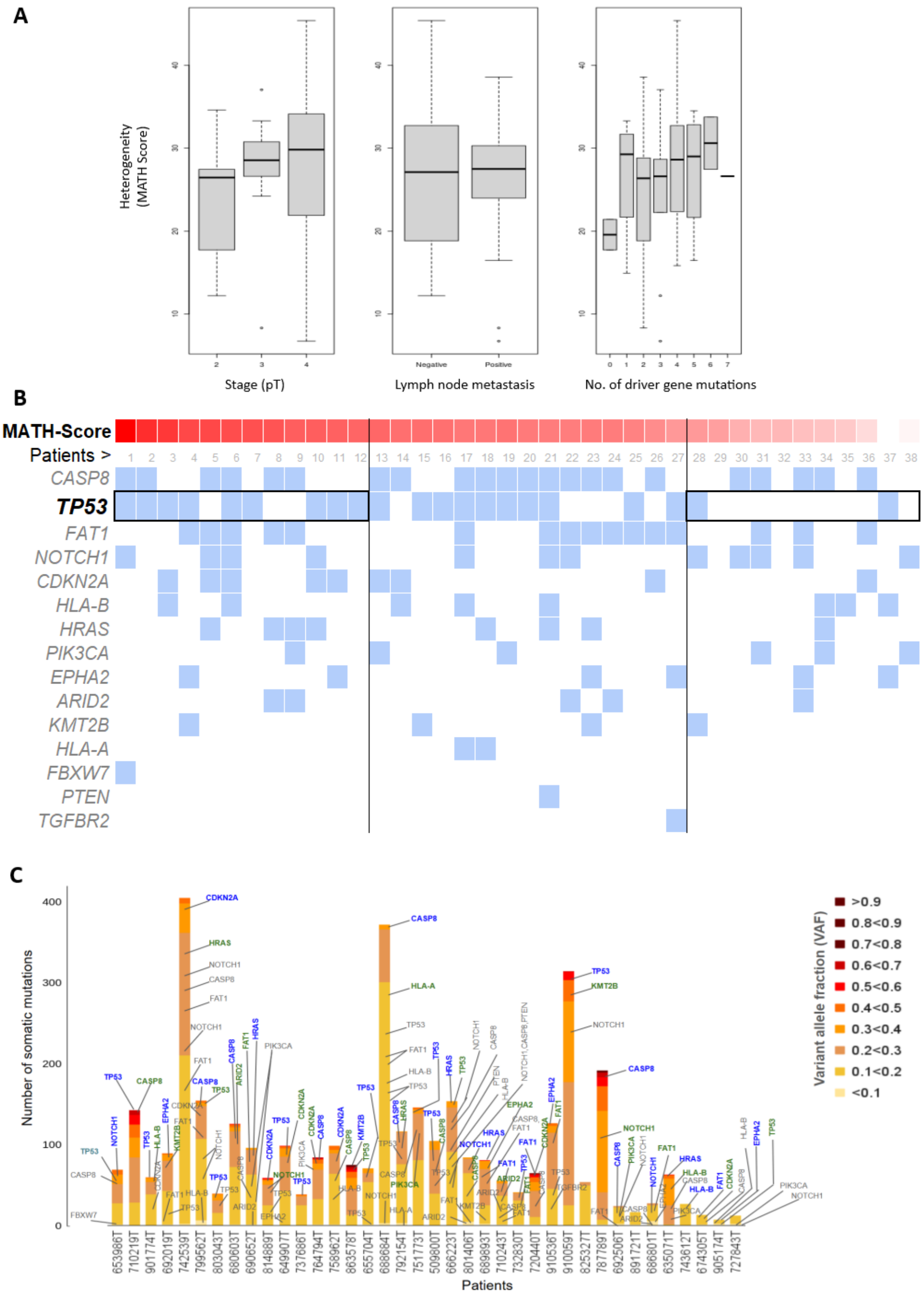

Supplement: Supplementary file 1 — Supporting Information [file CTM2-15-e70386-s002.pdf]
