# Supplementary material for: Comprehensive genomic and digital pathology profiling of tobacco‐chewer female oral cancer patients simultaneously with integration of single‐cell datasets identifies clinically actionable patient subgroups
Source: Clin Transl Med. 2025 Jul 7;15(7):e70386. doi: 10.1002/ctm2.70386 (PMC12230625; doi:10.1002/ctm2.70386)
Supplement: Supplementary file 2 — Supporting Information [file CTM2-15-e70386-s008.pdf]

Supplementary methods

Comprehensive genomic and digital pathology profiling of tobacco-chewer female oral cancer patients simultaneously with integration of single-cell datasets identifies clinically actionable patient subgroups

Arnab Ghosh[1,2,┼], Siddharth Singh[3,4,┼], Tuneer R Mallick[1,2], Shouvik Chakravarty[1,2], Supriya Varsha Bhagat[3], Chitrarpita Das[1], Kodaganur S Gopinath[5], Azeem Mohiyuddin[5], Arindam Maitra[1], Partha P Majumder[6,7,*], Tapas K Kundu[3,*] and Nidhan K Biswas[1,*]

[1]Biotechnology Research and Innovation Council-National Institute of Biomedical Genomics (BRIC-NIBMG), Kalyani, India
[2]Biotechnology Research and Innovation Council-Regional Centre for Biotechnology (BRIC-RCB), Faridabad, India
[3]Molecular Biology and Genetics Unit, Jawaharlal Nehru Centre for Advanced Scientific Research, Bangalore 560064, India
[4]Laboratory of Signaling and Gene Regulation, Cecil H. and Ida Green Center for Reproductive Biology Sciences, University of Texas Southwestern Medical Center, Dallas, Texas.
[5]Sri Devaraj Urs Academy of Higher Education and Research (SDUAHER), Kolar, India
[6]John C. Martin Centre for Liver Research and Innovations, Kolkata, India
[7]Indian Statistical Institute, Kolkata, India

┼ A Ghosh and S Singh contributed equally.

*** Corresponding authors**

Nidhan K Biswas, PhD
Biotechnology Research and Innovation Council-National Institute of Biomedical Genomics (BRIC-NIBMG), Kalyani-741251, India
E-mail: nkb1@nibmg.ac.in

Partha P Majumder, PhD
Indian Statistical Institute, Kolkata-700108, India
Email: parmaj2023@gmail.com

and

Tapas K Kundu, PhD
Molecular Biology and Genetics Unit, Jawaharlal Nehru Centre for Advanced Scientific Research (JNCASR), Jakkur, Bangalore-560064, Karnataka, India
E-mail: tapas@jncasr.ac.in

Patient recruitment, demographics and biospecimen collection

To characterize the oral somatic mutational landscape among female patients from the south India, a collaborative study funded by DBT, Govt of India, was undertaken by scientists of two national institutions in India (JNCASR, Bengaluru and NIBMG, Kalyani). We recruited 38 treatment-naive patients from R.L. Jalappa Hospital & Research Centre, Tamaka, Kolar. Institutional ethics approval was obtained from the hospital, and all study participants were recruited after obtaining voluntary signed informed consent. The patients were aged between 40 and 76 years (mean = 55.06 years) and diagnosed with gingivo-buccal oral cancer, of whom 55.26% were diagnosed at pathologically advanced stages III & IV. Lymph node metastasis was detected for 42.10% patients most of whom (68.42%) were at an advanced stage. All the patients were habitual tobacco chewers, but not smokers. Tissue from the tumour and a 5mL blood sample were collected from each patient during surgery. Post-surgery, patients received radiation therapy (RT) with (63.16%) or without (28.95%) adjuvant chemotherapy (CT). The median disease-free survival (DFS) was 28 months [range 9-38 months] during a follow-up period of 3-4 years. Detailed clinical information is provided in **Table S1**.

Whole exome sequencing, data QC and detection of somatic mutation

DNA was isolated from paired tumour tissue and blood of 38 patients using Sigma-Aldrich GenElute Mammalian Genomic DNA Purification Kit (G1N70). The concentration of DNA was measured using NanoDrop 2000 and OD260/280 was > 1.8 for each sample. About 45 Mb of coding region of the human genome was captured using TruSeq exome enrichment kit for WES library preparation followed by paired-end sequence data generation using Illumina NovaSeq6000 at about 100x depth of coverage. Only high-quality paired-end reads with 90% bases with quality value > 20, < 5% N content and no adapter content (determined by trimmomatic-0.27[1]) were retained for downstream analysis followed by standard QC assessment using FastQC-0.11.7[2]. The reads were aligned with human reference genome (hs37d5) using BWA-MEM[3](default parameters, with additionally, -M, -T 1) followed by removal of optical duplicates (PICARD-2.17.11[4]), local indel realignment and base quality score recalibration (GATK-3.8[5], with known 1000Genomes, dbSNP, Hapmap and Mills and 1000G gold standard indel sites). Further, unmapped, multi-mapped and reads with low mapping quality i.e., < 40 were filtered out through samtools-1.8[6]. The coverage and various other QC parameters were evaluated with Qualimap-2.2.1 tools[7]; a median of 89.22x and 94.51x depth of coverage was obtained for blood and tumour samples respectively. The cross-sample contamination was evaluated through GATK CalculateContamination with gnomAD

population sites prior to variant calling. Somatic alterations in tumours were detected by GATK-4 Mutect2[5] using blood WES data as normal. Somatic alterations that were not validated by any one of the other variant calling algorithms (MuSE[8] and Strelka-2.8.4[9]) were filtered out. Variants detected as Oxo-G artifact[10] through utilizing pair-orientation-bias information were filtered out. Variants lacking support from both DNA strands, encompassing low complexity region of genome (variant base homopolymer > 5, reference spanning +50bp region mapped to multiple locations with 98% sequence similarity by ncbi-blast-2.7.1[11]) were removed. Further somatic alterations with (a) depth of coverage > 10 in tumour and > 8 in blood, (b) variant allele count in tumour > 3 and 0 in blood, and (c) variant allele frequency in tumour > 0.1 were selected. Functional annotations for these somatic variants were generated using Oncotator-1.9.9.0[12] with the most recent Oncotator database. To remove germline artifacts from the somatic alteration list, we removed variants with population allele frequency > 0.01 in any subpopulation of 1000G[13] or GenomeAsia100K[14] and variants present in panel of normal (constructed from joint genotyping with all blood samples through GATK-4 HaplotypeCaller[15]). Variants residing in non-coding regions (off-targets) were removed from the somatic variant call set. Significantly mutated genes in this cohort were identified by MutSig2CV[16] algorithm (q < 0.1). All somatic non-silent mutations detected in previously known oral cancer or head and neck cancer driver genes were further manually verified through IGV.

Mutation signature analysis

The contributions of different mutagenic processes (as described in COSMICv3) on individual tumours were identified by analysing tri-nucleotide contexture around the mutated nucleotide through SigProfilerAssignment tool[17]. For each tumour, the contribution of each detected mutational signature (single base substitution signatures: SBS) and the cosine-similarities of the observed and matched models were computed by the algorithm encoded in the package[17] to infer relative contributions of mutational signatures.

Detection of somatic copy number alterations

Somatic copy number alterations (sCNA) were detected from each tumour samples through Illumina genome-wide genotyping array data (Infinium omni 2.5 v4 with 2.5 Million probes). The raw signal intensities from the image file were obtained through Illumina GenomeStudio followed by segmentation with ASCAT-2.5[18] package. The significantly altered somatic genomic regions were detected through GISTIC-2.0[19] package. Both focal and arm-level

amplifications and deletions were detected for individual tumours and annotated. Focal copy number amplification or deletions are considered at GISTIC-peak level with altered segment length not more than 2Mb.

Estimation of intra-tumour mutational heterogeneity

Variant allele frequency (VAF) estimation is a well-accepted proxy method for the timing of somatic mutational events, with a high variant allele fraction often indicating early origin for that particular mutation during the evolution of the tumour. We calculated VAF for each somatic mutation as the ratio of number of variant reads at the specific locus and total read coverage at that locus. Further, to understand mutational heterogeneity of the oral tumours, we calculated the widely used MATH (Mutant-allele tumour heterogeneity) score[20] for each individual tumour using the R package Maftools[21]. For a tumour-specific loci, MATH score is estimated from the median of variant allele fractions of those loci and median absolute deviation (MAD) of the variant allele fraction distribution, i,e., MATH = 100*MAD/median of variant allele fractions.

Pathway enrichment analysis

Pathway analysis was performed using somatic mutation data to understand the effect of mutations that influence specific biological processes. ReactomeFiviz plugin[22] of Cytoscape software[23] was used and pathway analysis was done implementing functional interaction (FI) models. Reactomefiviz makes use of a functional interaction database constructed from multiple data sources, such as - pathway level gene interactions from Reactome, KEGG and other repositories, known protein-protein and domain-domain interactions from databases and text mining, Gene ontology annotations, etc. Naive Bayes classifier algorithm is then used to identify high-confidence functional interactions. For our analysis we considered only those genes whose non-silent, coding mutations were observed in at least 3 patients. We have generated FI networks and further clustered them into modules, with each module having mutated genes over-represented in the cohort. FI modules have been annotated for pathway enrichment only if they contain at least 4 genes that are mutated (module size >= 4). Further, stringent statistical cutoff criteria (False discovery rate < 0.05) were implemented to identify pathways which were enriched with mutations.

Hematoxylin and Eosin staining of tumour tissue section

The collected tumour tissue samples were formalin-fixed and paraffin blocks were prepared. The sections from the tissues were characterized histopathologically by hematoxylin and eosin (H&E) staining. First, the tumour tissue sections were de-paraffinized in xylene for 5 minutes (3 times), followed by two changes in isopropyl alcohol for 5 minutes. The sections were stained with Delafield's hematoxylin (Sigma-Aldrich) for 10 minutes at room temperature and were rinsed in running tap water. Next, 1% acid alcohol was used to differentiate the tissue. In the blueing step, the tissues were washed well in tap water for 5 minutes. Finally, staining was performed with Eosin Y ethanol solution (Sigma-Aldrich) for 10 seconds. The sections were then dehydrated in isopropyl alcohol, cleared in xylene and mounted in DPX solution. The images from H&E slide sub sections were taken in Leica DMi8 M inverted microscope at 10X magnification. Whole slide imaging (WSI) data at 20X magnification was generated through Olympus IX85 microscope through scanning all H&E-stained tissue slides.

Quantification of tumour infiltrating leukocyte (TIL) from whole slide imaging data from H&E-stained slides

We have utilized a recently developed InceptionResNetV2 model through TILScout package (Zhang *et al*., 2025)[24] to quantify the proportion of tumour infiltrating leukocytes (TILs) from the 20X WSI scanned slides. Additionally, we have utilized digital pathology software QuPath (version 0.5.0)[25] to process the H&E image subsections to validate TIL infiltration at cell level. Through the cell detection module (with default parameters), the cells were detected from the H&E image from tumour sub-sections identified by pathologists. In order to detect the tumour infiltrating leukocyte (TIL), we have manually trained the object classifier (Random forest) with representative TIL (5 samples – yellow squared box) and other non-TIL cell populations (19 samples – red squared box) from one image (the field 1 of section from patient ID 799562) (identified by trained pathologist) as shown in Supplementary figure 1A to roughly classify TIL and non-TIL populations of cells. In order to achieve better classification (reduction of false positive detection), we have incorporated several heterogeneous representations of non-TILs segments while training the classifier. Finally, the trained model was applied for all H&E images from 15 patients (representative of major molecular sub-groups; at least two fields from tumour core sections from each patient) and the identified cells were classified into TIL (yellow) and non-TIL (red) groups. Further the proportion of TIL-classified cells to the total number of detected cells were calculated for each image. Finally, the average proportion of TILs from all images from a single patient was considered as the proportion of TILs in tumour

of that patient. The representative image for tumours detected with high and low TILs are shown in **Figure S5B and C**. See Figure S5D for comparison of segment level and cell level detection of TILs.

Single-cell RNAseq data processing, integration and cell type identification

We obtained single-cell transcriptome profiling data for 273,965 cells from 101 samples with head and neck cancers published by studies (GSE103322: n = 5902 cells, GSE164690: n = 48,868 cells, GSE182227: n = 70,970 cells, GSE181919: n = 54,239 cells, GSE215403: n = 52,393 cells; GSE172577; n = 65,255 cells)[26–30]. We used the Seurat (version 5.1.0) package [31] to process and analyse the publicly available scRNAseq datasets. Raw gene expression counts were converted into Seurat[31] object using ReadMtx command and loaded into R package. Poor quality cells were filtered out based on the number of genes expressed, mitochondrial reads, ribosomal reads and number of UMIs per cell. Doublets were removed using R package scDblFinder[32]. 138,527 cells passing QC criteria across five studies were merged into a single SeuratObject with multiple layers. Data in each layer was normalised using the LogNormalize method with a scale factor of 10,000. Top 2000 genes variably expressed in each layer were identified using the "vst" method. Normalised expression values for all genes were then scaled after regressing out the effects of mitochondrial reads, ribosomal reads and number of UMIs. Single cell data was further integrated using community approved protocol by removing study specific effects. Cells were classified into 3 broad cell lineages: 1) Epithelial (CDH1 and EPCAM positive), 2) Stromal (VIM positive), and 3) Immune (PTPRC, also known as CD45). We further classified immune cells to 12 immune cell types, including, CD8+ T cell, CD4+ T cell, NK cells, Treg cells, gamma-delta T cells, B cells, plasma cells, monocytes and DCs, macrophages, and mast cells. Dendritic cells were further classified into 1) conventional DCs (cDC) and 2) plasmacytoid DCs (pDCs) based on markers CCL22 and LAMP3 for cDC and TCF4, IRF7 and PLAC8 for pDC. Cell type annotation was performed based on markers described by previous studies[27] as well as existing databases[32,33]. For downstream analysis, 66,809 cells from 55 oral cavity tumours (excluding oropharynx and larynx tissue from the integrated head and neck data) were selected. Gross copy number profiles for genes at patient level were estimated using CopyKAT[34] tool from scRNAseq data.

Epithelial and immune cell interaction analysis

To infer cell-cell communication based on known ligand-receptor interactions between epithelial and immune cell types, we used the CellChat[35] (version 2.1.2) package in R. The

normalised counts matrix was loaded along with the cell type annotations into a ‘CellChat’ object. The data was pre-processed using commands, such as, identifyOverExpressedGenes (genes expressed in at least 30% cells and log2FC $\geq$ 0.25), identifyOverExpressedInteractions, using the ‘Secreted Signaling’ module in the ‘CellChatDB.human’ database. Probabilities of ligand-receptor interactions were calculated using the computeCommunProb, computeCommunProbPathway and aggregationNet functions. Communications in less than 10 cells in each group were filtered out. Interactions were visualised using netVisual_bubble functions. To investigate the downstream effects of ligand-receptor interactions we used NicheNet[36] (version 2.1.5). We obtained a list of potential ligands overexpressed in at least 10% of EGFR-High epithelial cells with average log2FC $\geq$ 0.25 and BH-adjusted p-value less than 0.05, compared to EGFR-Low epithelial cells. We designated a list of genes overexpressed in CD8 T cells of EGFR-High tumours as our geneset of interest. We used NicheNet to evaluate the weight of these ligand-receptor interactions.

Tissue Lysate Preparation and Western Blotting

Whole-cell proteins were extracted from the patient tissue samples (stored in PIC) in RIPA lysis buffer (50 mM Tris HCl [pH 8.0], 125mM NaCl, 1 mM EDTA, 0.5% NP-40, 1 mM dithiothreitol, 1mM phenylmethylsulfonyl fluoride) containing protease inhibitors. Samples were homogenized using mortar and pestle and kept in end-to-end rotor for 3 hours in cold room (4˚ C). Homogenized samples were centrifuged for 20 mins at 12,000 rpm and 4˚ C. The supernatant was collected in fresh microcentrifuge tubes and the protein content was determined using Bradford method. For future use, the samples were aliquoted and stored at -80˚ C after flash freeze.

Protein samples were separated on SDS-polyacrylamide gel electrophoresis (SDS-PAGE) gel and electrophoresed. Higher molecular weight proteins were transferred to the PVDF membrane at 25V for 50 minutes. The blots were blocked for an hour with 5% skimmed milk and probed with mouse primary antibody against Caspase-8 (1:1000), HRP-conjugated Beta-Actin antibody (1:2500) overnight at 4˚ C. Blots were washed 3 times with PBS (phosphate buffer saline) before being probed with HRP-conjugated secondary antibody (1:10000) for 2 hours at room temperature. The blots were washed 3 times with PBS and developed in Bio-Rad gel documentation system using electrochemical luminescence (ECL) western clarity solution.

References

1. Bolger, A. M., Lohse, M. & Usadel, B. Trimmomatic: A flexible trimmer for Illumina sequence data. *Bioinformatics* **30**, 2114–2120 (2014).
2. Andrews, S. & others. FastQC: a quality control tool for high throughput sequence data. Preprint at (2010).
3. Li, H. Aligning sequence reads, clone sequences and assembly contigs with BWA-MEM. Preprint at http://arxiv.org/abs/1303.3997 (2013).
4. Broad Institute. Picard Tools. *Broad Institute, GitHub repository* version 2.18.2 Preprint at http://broadinstitute.github.io/picard/ (2018).
5. McKenna, A. *et al.* The genome analysis toolkit: A MapReduce framework for analyzing next-generation DNA sequencing data. *Genome Res* **20**, 1297–1303 (2010).
6. Li, H. *et al.* The Sequence Alignment/Map format and SAMtools. *Bioinformatics* **25**, 2078–2079 (2009).
7. García-Alcalde, F. *et al.* Qualimap: Evaluating next-generation sequencing alignment data. *Bioinformatics* **28**, 2678–2679 (2012).
8. Fan, Y. *et al.* MuSE: accounting for tumor heterogeneity using a sample-specific error model improves sensitivity and specificity in mutation calling from sequencing data. *Genome Biol* **17**, 178 (2016).
9. Kim, S. *et al.* Strelka2: fast and accurate calling of germline and somatic variants. *Nat Methods* **15**, 591–594 (2018).
10. Costello, M. *et al.* Discovery and characterization of artifactual mutations in deep coverage targeted capture sequencing data due to oxidative DNA damage during sample preparation. *Nucleic Acids Res* **41**, e67–e67 (2013).
11. Altschul, S. F., Gish, W., Miller, W., Myers, E. W. & Lipman, D. J. Basic local alignment search tool. *J Mol Biol* **215**, 403–410 (1990).
12. Ramos, A. H. *et al.* Oncotator: Cancer variant annotation tool. *Hum Mutat* **36**, E2423–E2429 (2015).
13. Auton, A. *et al.* A global reference for human genetic variation. *Nature* **526**, 68–74 (2015).
14. Wall, J. D. *et al.* The GenomeAsia 100K Project enables genetic discoveries across Asia. *Nature* **576**, 106–111 (2019).
15. Garrison, E. & Marth, G. Haplotype-based variant detection from short-read sequencing. (2012).
16. Lawrence, M. S. *et al.* Discovery and saturation analysis of cancer genes across 21 tumour types. *Nature* **505**, 495–501 (2014).
17. Díaz-Gay, M. *et al.* Assigning mutational signatures to individual samples and individual somatic mutations with SigProfilerAssignment. *Bioinformatics* **39**, btad756 (2023).
18. Van Loo, P. *et al.* Allele-specific copy number analysis of tumors. *Proc Natl Acad Sci U S A* **107**, 16910–16915 (2010).
19. Mermel, C. H. *et al.* GISTIC2.0 facilitates sensitive and confident localization of the targets of focal somatic copy-number alteration in human cancers. *Genome Biol* **12**, R41 (2011).

20. Mroz, E. A. & Rocco, J. W. MATH, a novel measure of intratumor genetic heterogeneity, is high in poor-outcome classes of head and neck squamous cell carcinoma. *Oral Oncol* **49**, 211–215 (2013).
21. Mayakonda, A., Lin, D.-C., Assenov, Y., Plass, C. & Koeffler, H. P. Maftools: efficient and comprehensive analysis of somatic variants in cancer. *Genome Res* (2018).
22. Wu, G., Dawson, E., Duong, A., Haw, R. & Stein, L. ReactomeFIViz: a Cytoscape app for pathway and network-based data analysis [version 2; peer review: 2 approved] . *F1000Res* **3**, (2014).
23. Shannon, P. *et al.* Cytoscape: A Software Environment for Integrated Models of Biomolecular Interaction Networks. *Genome Res* **13**, 2498–2504 (2003).
24. Zhang, H. *et al.* Prediction and analysis of tumor infiltrating lymphocytes across 28 cancers by TILScout using deep learning. *NPJ Precis Oncol* **9**, 76 (2025).
25. Bankhead, P. *et al.* QuPath: Open source software for digital pathology image analysis. *Sci Rep* **7**, 16878 (2017).
26. Kürten, C. H. L. *et al.* Investigating immune and non-immune cell interactions in head and neck tumors by single-cell RNA sequencing. *Nat Commun* **12**, 7338 (2021).
27. Puram, S. V *et al.* Cellular states are coupled to genomic and viral heterogeneity in HPV-related oropharyngeal carcinoma. *Nat Genet* **55**, 640–650 (2023).
28. Choi, J.-H. *et al.* Single-cell transcriptome profiling of the stepwise progression of head and neck cancer. *Nat Commun* **14**, 1055 (2023).
29. Kurkalang, S. *et al.* Single-cell transcriptomic analysis of gingivo-buccal oral cancer reveals two dominant cellular programs. *Cancer Sci* **114**, 4732–4746 (2023).
30. Peng, Y. *et al.* Single-cell profiling of tumor-infiltrating TCF1/TCF7+ T cells reveals a T lymphocyte subset associated with tertiary lymphoid structures/organs and a superior prognosis in oral cancer. *Oral Oncol* **119**, 105348 (2021).
31. Hao, Y. *et al.* Dictionary learning for integrative, multimodal and scalable single-cell analysis. *Nat Biotechnol* **42**, 293–304 (2024).
32. Germain, P. L., Lun, A., Garcia Meixide, C., Macnair, W. & Robinson, M. D. Doublet identification in single-cell sequencing data using scDblFinder [version 2; peer review: 2 approved] . *F1000Res* **10**, (2022).
33. Program, C. Z. I. S.-C. B. *et al.* CZ CELL×GENE Discover: A single-cell data platform for scalable exploration, analysis and modeling of aggregated data. *bioRxiv* 2023.10.30.563174 (2023) doi:10.1101/2023.10.30.563174.
34. Gao, R. *et al.* Delineating copy number and clonal substructure in human tumors from single-cell transcriptomes. *Nat Biotechnol* **39**, 599–608 (2021).
35. Jin, S. *et al.* Inference and analysis of cell-cell communication using CellChat. *Nat Commun* **12**, 1088 (2021).
36. Browaeys, R., Saelens, W. & Saeys, Y. NicheNet: modeling intercellular communication by linking ligands to target genes. *Nat Methods* **17**, 159–162 (2020).
