# Supplementary material for: Comprehensive genomic and digital pathology profiling of tobacco‐chewer female oral cancer patients simultaneously with integration of single‐cell datasets identifies clinically actionable patient subgroups
Source: Clin Transl Med. 2025 Jul 7;15(7):e70386. doi: 10.1002/ctm2.70386 (PMC12230625; doi:10.1002/ctm2.70386)
Supplement: Supplementary file 3 — Supporting Information [file CTM2-15-e70386-s007.pdf]

Supplementary results

Comprehensive genomic and digital pathology profiling of tobacco-chewer female oral cancer patients simultaneously with integration of single-cell datasets identifies clinically actionable patient subgroups

Arnab Ghosh[1,2,┼], Siddharth Singh[3,4,┼], Tuneer R Mallick[1,2], Shouvik Chakravarty[1,2], Supriya Varsha Bhagat[3], Chitrarpita Das[1], Kodaganur S Gopinath[5], Azeem Mohiyuddin[5], Arindam Maitra[1], Partha P Majumder[6,7,*], Tapas K Kundu[3,*] and Nidhan K Biswas[1,*]

[1]Biotechnology Research and Innovation Council-National Institute of Biomedical Genomics (BRIC-NIBMG), Kalyani, India
[2]Biotechnology Research and Innovation Council-Regional Centre for Biotechnology (BRIC-RCB), Faridabad, India
[3]Molecular Biology and Genetics Unit, Jawaharlal Nehru Centre for Advanced Scientific Research, Bangalore 560064, India
[4]Laboratory of Signaling and Gene Regulation, Cecil H. and Ida Green Center for Reproductive Biology Sciences, University of Texas Southwestern Medical Center, Dallas, Texas.
[5]Sri Devaraj Urs Academy of Higher Education and Research (SDUAHER), Kolar, India
[6]John C. Martin Centre for Liver Research and Innovations, Kolkata, India
[7]Indian Statistical Institute, Kolkata, India

┼ A Ghosh and S Singh contributed equally.

*** Corresponding authors**

Nidhan K Biswas, PhD
Biotechnology Research and Innovation Council-National Institute of Biomedical Genomics (BRIC-NIBMG), Kalyani-741251, India
E-mail: nkb1@nibmg.ac.in

Partha P Majumder, PhD
Indian Statistical Institute, Kolkata-700108, India
Email: parmaj2023@gmail.com

and

Tapas K Kundu, PhD
Molecular Biology and Genetics Unit, Jawaharlal Nehru Centre for Advanced Scientific Research (JNCASR), Jakkur, Bangalore-560064, Karnataka, India
E-mail: tapas@jncasr.ac.in

Background

Oral cavity cancer, primarily caused by tobacco consumption is the most prevalent form of cancer among men and the sixth most common among women in India. The most affected subsite in India is the gingivo-buccal region, rather than the tongue, which is most frequently affected in populations of the global West. The somatic mutational landscape of oral gingivo-buccal squamous cell carcinoma (OSCC-GB) significantly differs from that of the tongue squamous cell carcinoma[1,2]. Being the most prevalent among males, there is paucity of genomic data on tumours from female OSCC-GB patients. The ICMR-NCDIR data[3] showed that the percentage of tobacco affected cancer sub-sites among south Indian females – female patients in this study were drawn from south India – is around 13% and 38.96% among males. The evidence of differential cancer susceptibility and survival based on ethnicity[4] and sex[5] have been shown previously. The clinical characteristics along with driver gene alteration frequencies for several cancer types (e.g., lung, colorectal, etc.) showed differences with respect to ethnicity (among African American, Caucasian and Asian)[4]. Specific polymorphisms in particular populations have been implicated in increasing cancer risk; for example, *TP53* codon 72 polymorphisms in Asians with gastric cancer[6] and in African Americans with colon cancer[7]. Sex differences influencing DNA repair[8], immune response[9],metabolism[10], etc. have been implicated in differential prevalence and outcome in cancer types.

Previously, multiple national and international consortia have performed whole-exome analyses in predominantly male oral and head and neck cancer patients to identify a number of alterations in driver genes such as *TP53*, *CASP8* and *NOTCH1*, and elucidate their role in tumour pathogenicity[1,2,11]. Under the aegis of the International Cancer Genome Consortium (ICGC), we have generated whole-exome sequence data from a large set of OSCC-GB patients from India[1]; 88% of the patients were male. Sex differences are known to impact on human health and disease including cancer[12]. Therefore, we considered it important to investigate sex differences in molecular genomic profiles of oral tumours.

In the present study [**Figure S1**], we have recruited 38 female oral cancer patients (including ~44% with early-stage tumours) from south India (Kolar district of Karnataka State) and generated somatic mutational and copy number alteration profiles by analyzing whole exome

sequencing and genomewide array data. We have identified 9 significantly mutated genes which were previously found to be drivers of oral cancer although the alteration frequencies for some of these genes significantly differed from male dominated oral cancer cohorts. We have shown that *TP53* mutational status is linked to greater tumour heterogeneity. We have performed integrative analysis of mutation and digital pathology image data to infer insights on immune infiltration. In addition, we have integrated publicly available single-cell gene expression datasets comprising 66,809 cells to identify plausible mechanisms for immune suppression in the context of genomic alterations found in this patient cohort. In summary, our analysis has identified two major subgroups of female oral cancer patients which shows potential to get benefit from available therapeutics.

Results

Somatic mutation and copy number landscape identify distinct patient subgroups

By analyzing WES data [**Supplementary data 1**] of OSCC-GB tumours from 38 female patients from southern India [**Table S1**], we detected a total of 3846 nonsynonymous and 1295 synonymous somatic mutations, with a median of 105.5 mutations per patient [range: 8-564, **Figure 1A** and **Table S2**]. Ten previously known OSCC-GB driver genes[1,13], *CASP8* (mutated in 57.89% patients), *TP53* (55.26%), *FAT1* (42.11%), *NOTCH1* (31.58%), *CDKN2A* (23.68%), *HLA-B* (21.05%), *HRAS* (21.05%), *EPHA2* (15.79%), *PIK3CA* (15.79%) and *ARID2* (13.16%) were found to be significantly mutated ($q < 0.1$, MutSig2CV algorithm) in this cohort [**Figure 1B** and **Supplementary data 2**]. Mutational distribution of the driver genes on the background of protein domains is described with corresponding onco-kb annotations in **Figure S2**. Somatic mutations in other previously known oral cancer driver gene *KMT2B* (10.52%) along with head and neck cancer driver genes[11] *HLA-A* (5.26%), *PTEN* (2.63%) and *FBXW7* (2.63%) were also found in this cohort. About 95% of the patients (36 of 38) harbour somatic mutations in at least one of these driver genes, and 94.44% (34) of these 36 patients either had *TP53* and/or *CASP8* alterations. For the remaining 2 patients in whose tumors, we did not detect somatic mutations in known oral cancer driver genes, but we found somatic mutations in other cancer driver (source: TCGA PanCan Atlas) genes[14] [*STAG2* was mutated in one patient and another harboured somatic mutation in *HUWE1*, *STK11*, *RRAS2*, and *SETD2* genes, see **Supplementary data 2**]. We identified 8 major pathway modules to be significantly mutated

- (i) RNA Polymerase II Transcription (q=1.65E-04), (ii) Notch signaling pathway (q=9.65E-06), (iii) PI3K-Akt signaling pathway (q=2.62E-05), (iv) Integrin signaling pathway (q=5.76E-06), (v) Calcium signaling pathway (q=1.96E-08), (vi) cAMP signaling pathway (q=2.28E-07), (vii) Wnt signaling pathway (q=2.71E-09), (viii) Cadherin signaling pathway (q=1.05E-09) (**Figure S8** and **Table S5**) [Analysis excluding known cancer drivers additionally showed Focal adhesion pathway to be significantly enriched (q=4.62E-04)].

A median of 3 oral cancer driver genes (range 0-7) were detected per patient. Many notable differences were found between male and female OSCC-GB patients. We observed that the mutation frequency of *TP53* (55.26%) in this female patient cohort is bit lower than among the male patients in ICGC-India OSCC-GB cohort (60.92%, N=87) [the somatic mutation data of OSCC-GB from ICGC cohort was obtained from dbGENVOC[15]], and it was significantly (*p*=0.0001, Fisher's exact test) lower than HPV- male patients of TCGA head and neck cancer cohort (84.93%) [**Table 1**]. On the other hand, the prevalence of *CASP8* (57.89%), *NOTCH1* (31.58%), *CDKN2A* (23.68%), *HRAS* (21.05%), *HLA-B* (21.05%) and *EPHA2* (15.79%) mutations were significantly higher (*p*<0.026, Fisher's exact test) in these patients than among male patients of ICGC-India OSCC-GB cohort (*NOTCH1*: 10.34%, *CDKN2A*: 5.75%, *HLA-B*: 6.90% and *EPHA2*: 2.30%) [**Table 1**]. Our previous study on whole exome sequencing of Indian OSCC-GB patients as part of ICGC[1] revealed two major clusters of patients – (a) with primarily *TP53* somatic mutations (39.09% patients), and (b) with *CASP8* mutations (31.82% patients) [of which 50% co-occurred with *TP53* mutations]; 69% of *TP53* mutations (50 of 72) occurred without *CASP8* mutation. Based on these prior, four broad molecular sub-groups were identified in the current cohort – patients with (1) both *CASP8* and *TP53* mutations (23.68% patients), (2) *CASP8* mutation without *TP53* mutation (34.21%), (3) *TP53* mutation without *CASP8* mutation (31.58%), and (4) Both *CASP8* and *TP53* wild-type (10.53%) [**Figure 1B**]. In this cohort, 57% of tumours with *TP53* mutations were observed without mutated *CASP8* [**Figure 1B**], which was less compared to ICGC-OSCC-GB cohort although not statistically significant (*p* = 0.1464, Chi-squared test). Patients with *TP35* mutations showed significantly (*p* = 0.035, Log-rank test) poorer disease-free survival (DFS) (median of 21 months) than those without *TP53* somatic mutations (32 months) [**Figure S4**]. We observed that 84.62% *CASP8* mutations were functionally pathogenic (i.e., nonsense, splice-site, insertion and deletion), when they do not co-occur with *TP53*, which was significantly higher (*p* = 0.007, Chi-square test) than when co-occurred with *TP53* mutations (33.33% of *CASP8* mutations are pathogenic when co-occurred with *TP53*). Among the 26 *TP53* mutations, 3 were detected to be known hotspot mutations which include p.T125T (splice site), p.R273C

(missense), and p.R213* (nonsense). The lymph node involvement was significantly higher (p = 0.0141, Chi-square test) among *TP53* mutated patients (57.14%) as compared to *TP53* wild type (25.92%). All detected *CDKN2A* mutations in this cohort were either splice site or nonsense mutations – indicating complete loss-of-function. 4 of 7 *PIK3CA* mutations (including p.E545K/Q, p.H1047R/L) were known oncogenic hotspots[16]. Alongside *PIK3CA*, nonsynonymous somatic mutations in other genes in the PI3K-AKT pathway - *PIK3CD*, *PIK3R2*, *PIK3AP1* and *PI3R4* were detected among these patients; they were present in 22.92% patients [**Figure 1B**]. Interestingly among the somatic mutations in *PIK3CA*, *PIK3CD*, *PIK3R2*, *PIK3AP1* and *PI3R4* genes, 90.91% (10 of 11) co-occurred with *CASP8* mutation. All *HRAS* somatic mutations were in codons 12 and 13 that are previously known oncogenic hotspots[17]. 11 of 38 patients (28.95%) in this cohort harboured oncogenic mutations in either *TP53* or *PIK3CA* and *HRAS* [**Figure 1B**]. As *CASP8* was the most frequently mutated in this cohort, we checked the protein level expression of caspase-8 in a subset (9) of patients' tumor samples harboring wild-type or varying *CASP8* mutations (**Figure S3**). Procaspase-8 expression was observed in all the samples. Interestingly, in all the three tumor samples with an N-terminal Caspase-8 mutation, expression of activated caspase-8 (in cleaved form) was observed. However, among the 5 tumor samples with C-terminal *CASP8* mutations, two tumors (harbouring truncating mutations p.E259* and p.G409*) did not show any cleaved caspase-8 expression (similar to the tumor with wild-type caspase-8). In a single tumor (with truncating mutation p.Q420*), lower expression of procaspase-8 and enhanced cleaved caspase-8 was observed (**Figure S3**).

The somatic copy number alterations (CNAs) in this cohort were determined by analyzing CNV-array data generated for 36 patients [array data for 2 patients could not be included because of inadequate quality.]; the median CNA burden (calculated as the percentage of genome amplified or deleted) was estimated to be about 0.09 [average = 1.1, range: 0.0016 - 23.5]. Chromosome arms – 3p (19.4% patients), 8p (13.9%), 8q (5.5%), 19p (16.7%) and 19q (11.1%) were significantly ($q < 0.1$, GISTIC) deleted and 3q (11.1%), 8q (19.4%), 9p (16.7%), 9q (13.9%), and 14q (11.1%) were significantly ($q < 0.1$, GISTIC) amplified in this patient cohort [**Table S3**]. Alongside arm-level alterations, focal amplifications and deletions were also detected. We found 4 genes to be significantly ($q < 0.1$, GISTIC) amplified that including *ALDH1L1* (amplified in 56.5% patients) and *EGFR* (17.4%), and 2 genes [including known fragile site[18] associated deletions in *FHIT* (deleted in 43.5% patients) and *CSMD1* (28.3%)] were significantly deleted in this cohort. We found *CDK7*, *CDKN2A*, *BRAF*, and *FAT1* to be

deleted in 33.3%, 25.0%, 13.9% and 8.3% patients. The *KCNJ11* gene was found to be amplified in 22.2% of patients.

Mutational signature analysis reveals unique histories of oral tumours

Tumours evolve through accumulation of somatic mutations with active involvement of several mutagenic processes. Such mutational signatures can be deconvoluted by studying trinucleotide contexts of each somatic mutation[19,20]. The trinucleotide context of each somatic point mutation was analyzed and compared with COSMIC (v3) database to detect mutational signatures pointing to mutagenic processes in this patient cohort [**Table S6**]. We detected 31 COSMICv3 signatures [**Figure 1C**], with 12 predominant signatures (present in $\geq$ 10% patients) – SBS1 (86.8%), SBS5 (84.2%), SBS2 (31.6%), SBS13 (21.1%), SBS4 (21.1%), SBS10b (15.8%), SBS15 (15.8%), SBS7a (15.8%), SBS87 (15.8%), SBS30 (10.5%), SBS58 (10.5%), and SBS94 (10.5%). Signatures SBS1/5, 4, 2/13, 7a/7b/7d that, respectively, represent tumour aging, tobacco smoking, APOBEC activity and exposure to UV were previously found in Indian OSCC-GB tumours. The mutational signature SBS24 which is caused by "exposure to aflatoxin"[21] was found in one patient in this cohort. Somatic mutational signatures related to APOBEC activity (SBS2 and 13) were detected in 12 patients with an average contribution of 28.10% to the total mutations. Among the subset of tumours (n=15) for whom H&E WSI data was analyzed and TIL proportions were estimated, we detected APOBEC signature in 50% (4 of 8) of high-TIL tumours (described earlier) in contrast to 28.57% (2 of 7) among low-TIL tumours (Table S4). This observation agrees with an earlier study that showed association of APOBEC mutagenesis with high immune infiltration in TCGA head and neck cancer cohort[22].

EGFR amplification is associated with poor immune infiltration

For a subset of patients (n=15) with distinct somatic alteration landscape (*CASP8* and *TP53* both mutated, n=5; *CASP8* mutated but *TP53* not mutated, n=3; *TP53* mutated without mutation in *CASP8*, n=6; no driver gene mutation, n=1), at least two fields from H&E-stained section of primary tumours were captured for deeper histopathological assessment [**Figure 2A**]. We have observed a very distinct histopathological profile for an EGFR-amplified tumour without having any other driver gene mutations [**Figure 2A**]. Utilizing recently developed AI-based tumour infiltrating leukocyte (TIL) quantification model embedded in TILScout, we have obtained the proportion of TILs from the WSI (20X) generated for the tumours [Table S4 and Figure S5]. We have detected a median of 4.84% TILs from WSI of these tumours, which ranged between 0 to 27.19% - segregating the patients into two broader groups – (a) low (TIL proportion range: 0 - 0.5%) and (b) high (range: 4.84 – 27.19%) TIL. In addition to the window based approach of TILScout, we have also trained and tested a model in QuPath for detection of leukocytes within sub-sections of the WSIs for validation at cell level [Figure S6A, B, C, and Figure 2B]. Among the somatic driver alterations, we found that the proportions of TIL in oral tumours were significantly negatively associated with amplification of *EGFR* ($p = 0.007$, Fisher's exact test) [**Table S4** and **Figure 2B**]; the average fraction of TILs in *EGFR*-amplified tumours was 0.17% (range: 0 – 0.5%) while the remaining tumours had an average of 12.5% TILs (range: 0.14 – 27.19%). This observation was further validated in TCGA-HNSC cohort, in which the extent of immune infiltration (in terms of Immune Score obtained through ESTIMATE algorithm, equivalent to proportions of TILs) in *EGFR* amplified tumours (n=53, mean Immune Score = 212.81) was significantly ($p = 0.00195$, *t*-test) lower compared to the remaining patients (n = 469, mean Immune score = 476.75) [**Figure S7**]. EGFR amplification has been recently shown to be associated with decreased immune infiltration within tumours in case of lung[23] and gastroesophageal adenocarcinoma[24]. Though the proportion of TILs in these tumours did not correlate with the total number of somatic mutations in them, we observed that oral tumours with low-TIL proportions acquired a median of 2 driver gene somatic mutations whereas tumours with high-TIL acquired a median of 4.5 driver gene mutations. Interestingly, we found that tumours with oncogenic mutational signature (described earlier in the Results section – known oncogenic *TP53*, *PIK3CA* or *HRAS* mutations) had slightly higher proportions of TIL (median = 9.98%) compared to tumours without the signature (median = 0.34%) [**Table S4** and **Figure 2B**].Consistent with our finding, TCGA-HNSC tumours harbouring oncogenic mutational signature showed significantly (p = 0,0106, *t*-test) higher Immune Score (mean = 581.65) than the remaining (400.27) [**Figure S7**].

One of the tumours that acquired both oncogenic mutation (in *TP53*) and *EGFR* amplification showed low proportion of TIL (0.07%) within tumour [**Table S4**].

Integration of single-cell gene expression data reveals immune evasive mechanisms in EGFR-high head and neck tumours

EGFR expression was significantly positively correlated (r=0.5, p < 2.2e-16, Pearson's correlation) with its genomic copy number status in TCGA-HNSCC cohort (**Figure S7B**), to further elucidate the role of EGFR amplification on the immune micro-environment, we integrated 6 publicly available single cell RNAseq (scRNAseq) datasets [Figure S9A] comprising 66,809 cells from 55 patients with oral cavity cancers without HPV involvement. Based on cell lineage specific markers, we have initially stratified the cells into – (1) epithelial (CDH1+, EPCAM+), (2) stromal (VIM+), and (3) immune cells (CD45+) [**Figure 3A**]; followed by deeper stratification of immune cells into 12 subtypes - CD8+ T, CD4+ T, NK, Treg, gamma-delta T, B, plasma, monocytes and dendritic cells, macrophages, and mast cells [**Figure 3B**] – based on expression of known markers [**Figure S9B, C**]. Based on average EGFR expression in epithelial cells for each patient (estimated through pseudo-bulk approach implemented in Seurat), we have stratified patients into two stringent groups – (a) Group 1: EGFR-high (expression $\geq$ Q3; n=10), and (b) Group 2: EGFR-low (expression $\leq$ Q1; n=10) [**Figure 3E**]. Copy-number estimation from scRNAseq through CopyKAT predicted copy-number gains in 70% in high EGFR expressing tumours, which further confirms its copy number gain driven expression profiles [Figure S9D]. In line with our findings from integrative analysis of bulk genomic and digital pathology data, we detected reduced (1.71-fold) infiltration of TILs (comprising CD8+ T, CD4+ T, Tregs, gamma-delta T, NK, and B cells) in EGFR-high tumours (median proportion of TILs with respect to total number of TILs and malignant epithelial cells = 55.1%) than EGFR-low tumours (32.2%) single cell profiles [**Figure 3C**]. All subtypes of TILs showed a downward trend in infiltration in EGFR-high tumours [**Figure 3D**]. These results reflect an immune-depleted microenvironment in EGFR-high tumours. To generate further insight on plausible mechanisms of EGFR-expression driven immune suppression, we identified differentially expressed genes in EGFR-high versus low epithelial cells [**Figure 3F** and **Table S7**]. Key genes involved in antigen-presentation, including, multiple MHC class-I (*HLA-A*, *B* and *C*) and MHC class-II (*HLA-DRA*, *DRB1*, *DPA1*, and *DQB1*) molecules (average log2FC=-2.82) along with *B2M* (log2FC=-2.14) were significantly (q < 0.0001) downregulated in EGFR-high tumours compared to EGFR-low

tumours. Downregulation of antigen-presentation has been shown to be a hallmark of immune-evasive nature of tumours shown in multiple cancer types including lung, kidney etc. Several genes known to interact with EGFR – e.g., *ALCAM*, *NTRK2*, *EXOC3*, *IGFBP2*, *GJA1* (interaction analysis - STRINGdb) were significantly upregulated in EGFR-high epithelial cells than EGFR-low cells. In addition, genes like, *GPX2* which was previously found to be associated with immune cold nature of tumours[25] was significantly upregulated in EGFR-high tumours as compared to EGFR-low along with some of its interacting partners *AKR1C1*, and *AKR1C2* (interaction analysis - STRINGdb). *CXCL17*, *LCN2*, and *SAA1* genes which are involved in stimulating immune response were significantly downregulated in EGFR-high epithelial cells compare to EGFR-low. Additionally, EGFR-high tumours expressed significantly ($p < 0.0001$) high levels of pro-angiogenic genes such as *VEGFB* and *FGFBP1* (average log2FC=0.71) than EGFR-low tumours. In summary, multiple immune stimulating factors were found to be downregulated in EGFR-high epithelial cells which might be associated with low immune cell infiltration within the tumour microenvironment. Overall, the data shows an immune evasive and pro-angiogenic nature of epithelial cells in EGFR-high tumours which may contribute to low immune infiltration and aggressiveness. These features provide refined deep evidences to our findings based on bulk exome and digital pathology based interpretations.

Further, we identified differential receptor-ligand interactions of malignant epithelial cells with immune cells between EGFR-high and low tumours [**Figure S9E and F**]. Epithelial cells in EGFR-high tumors showed higher number of outgoing (epithelial to immune) interactions (102 interactions with $p < 0.05$) compared to EGFR-low tumours (75), indicating their role as potent regulators of the tumor microenvironment [**Figure S9G**]. Ligands like, PTN (interacting with NCL on immune cells), PLAU (interacting with PLAUR on macrophages, monocytes, and mast cells), and ADM[26] (interacting with CALCRL on pDCs) were significantly upregulated in EGFR-High epithelial cells, while MIF (interacting with CXCR4, CD74, and CD44[27–29]), PPIA (interacting with BSG) and CXCL16 (interacting with CXCR6 on CD8+ T, and Treg cells) were significantly upregulated in EGFR-Low epithelial cells [**Figure 3G** and **H**]. We noted that in case of EGFR-high tumors, CD8+ T cells had higher number of incoming interactions from other cells (76 active interactions with $p < 0.05$), compared to EGFR-low tumors (44 interactions). Key interactions like PTN (expressed in epithelial cell) - NCL (expressed in CD8+ T cell) and PROS1 – AXL were significantly enriched in EGFR-high tumours [**Figure S9H**]. Pleiotrophin (PTN) has been linked with increased tumor growth and angiogenesis in breast cancer[30]. Tumor cell-derived PROS1 expression has been linked with

impaired M1 macrophage polarization, thus leading suppression of anti-tumor immunity[31]. These results indicate an active immune remodelling of CD8+ T cells by EGFR-High epithelial cells. To assess the downstream effects of these interactions between epithelial and CD8+ T cells in EGFR-High tumors, we identified multiple ligand-receptor interactions between EGFR-high epithelial cells with CD8+ T cells, and their effects on downstream signalling in context to EGFR expression [**Figure 3I**]. Multiple collagen molecules[32] showed strong interaction potential with ITGA1 and ITGB1 of CD8+ T cells in EGFR-high tumours [**Figure 3J**]. We have observed EGFR-high epithelial cells significantly overexpress (log2FC > 0.25, q < 0.05) ligands like, LGALS3, CD59, MDK, IL8 etc. as compared to EGFR-low cells which are known to influence T cell interaction [**Figure 3J**]. Epithelial-derived ligand interaction with CD44[33] receptor on T cells may contribute to deficiency of CD8+ T cell activation [**Figure 3J**]. LGALS3 known to interact with LAG3, ANXA2[34], and MCAM[35]. Both LAG3 and ANXA2 are previously associated with immune suppression[36] through promoting T cell exhaustion and evasion of apoptosis [**Figure 3K**]. Overall, our integration of single-cell datasets further provided deeper insights into plausible immune suppression mechanisms in EGFR-positive tumours which is detected in almost one-fourth of patients in this cohort.

Two actionable molecular subgroups among Indian female oral cancer cohort

Joint analysis of genome alteration and digital pathology data with further integration of publicly available single cell gene expression datasets identified two major actionable subgroups within Indian female oral cancer cohort – patients harbouring (a) EGFR-amplification (22.22% of patients), and (b) oncogenic somatic mutations (31.58%). Tumours of 50% patients (18 of 36 for which both WES and CNV-array data was available) in this cohort carry at least one of the above two molecular features. Currently EGFR-positive OSCC tumours are treated with cetuximab[37] which has shown to improve on overall survival of the patients. Analysis of our current study additionally showed immune suppressive phenotype of EGFR-high tumours and we identified several receptor-ligand interactions (between tumour and immune cells) such as LGALS3-LAG3, PLAU – PLAUR, MDK – (ITGA4+ITGB1), for multiple of which inhibitors are already FDA-approved (Relatlimumab targeting LAG3[38,39]) or in clinical trial [Upamostat[40] targeting PLAU; Natalizumab targeting MDK[41,42]. On the other hand, a major fraction of patient, harbours *PIK3CA* / *HRAS* mutation for which inhibitors [Alpelisib[43], tipifarnib[44,45] for *PIK3CA* and *HRAS* mutation] already showed promise in clinical

trials. Additionally, our data showed high immune infiltration in these oral tumours with oncogenic mutations, which can have improved outcome through treatment in combination with neoadjuvant immune-checkpoint inhibitors[46]. In summary, our data identified plausible therapeutic targets for about 50% patients in female oral cancer cohort.

Discussion

This study has identified the somatic mutational and copy number alteration landscapes of female oral cancer patients from south India and identified molecular subgroups associated with differential disease progression. Globally, the prevalence of oral cancer is low among females. Therefore, female patients are under-represented in earlier studies that have sought to catalogue the somatic mutation landscape of oral cancer. We have shown that this female patient cohort had similarities of mutation profiles with those observed in previous studies from India[1] and the USA[11]. However, this female patient cohort has distinctive molecular subgroups. Integration of the tumour pathology data (H&E whole slide image) with the somatic mutational landscape and further integration of available single-cell data provided valuable insights on disease progression and opened up the possibility of immunotherapy in a large subset of patients.

In contrast to our previous study[13,47] on oral cancer patients from other cohorts in India, primarily from east and west India, the oral cancer patients in this cohort did not harbour excess of rare germline non-silent alterations in DNA repair pathways. We also did not detect HPV integration from the exome sequence data in any of the female patients. We note that HPV involvement in 3.5% of female patients has been observed in TCGA head and neck cancer cohort. The somatic mutational landscape revealed that *CASP8* gene was the most frequently mutated (57.89%) gene among the female oral cancer patients followed by *TP53* (55.26%); although *TP53* has been found to be the most frequently mutated genes in other gingivo-buccal oral cancer cohorts within India and outside (frequency range: 62-74%)[1,11,15]. In addition to *CASP8*, we found significantly high frequency of mutations of four genes – *NOTCH1*, *CDKN2A*, *HRAS*, *HLA-B* and *EPHA2* in this cohort compared to male patients included in the ICGC-India cohort. Somatic mutations of known oral cancer driver genes were found in almost all patients (37 out of 38) and the patient who did not acquire these driver gene somatic mutations, acquired copy number amplification in *EGFR*. *TP53* somatic mutation significantly reduced the duration of disease-free survival of these patients, suggesting that the presence of *TP53* mutation is a prominent prognostic factor in line with several past studies on oral[1] and other cancer types[48,49]. We found that a subset of patients (31.6%) acquired oncogenic mutations in *TP53*, *HRAS* or *PIK3CA*. Broadly we observed four groups of patients: (1) with both *CASP8* and *TP53* mutated [23.68% patients], (2) *CASP8* mutated without *TP53* mutation [34.21%], (3) *TP53* mutated without *CASP8* mutation [31.58%] and (4) with both *TP53* and

CASP8 wild-type with other or no driver gene mutation [10.53%]. Among *CASP8* mutations, 36.36% affected death-effector-domain (DED) and 59.09% affected peptidase domain. We observed 84.61% non-silent mutations in *TP53* gene occurred in the DNA binding domain (DBD), including three previously known hotspot amino acid 125, 213 and 273; the somatic mutation p.R273C found in a patient also has been previously characterized for oncogenic functionality[49] in tumourigenesis. Also, one of the non-hotspot *TP53* mutation p.P152L detected in a patient, has been shown to display several oncogenic properties, including enhanced tumorigenic potential in our former study[50]. A large fraction (42.31%) of *TP53* non-silent mutations in this cohort were stop-gain (i.e., premature stop codon), frameshift or splice-site mutations, indicating – loss-of-function (LoF) of *TP53*-driven tumourigenesis in these patients. The proportion of tumours with *EPHA2* alterations was significantly high in this cohort compared to previously reported OSCC data from India and other regions. Both tumour suppressive and oncogenic roles of *EPHA2* through its canonical and non-canonical downstream activity have been reported[51]; in our cohort, all *EPHA2* mutations were truncating (stop-gain and splice site) – therefore, loss-of-function of *EPHA2* in oral tumourigenesis is evident. Patients with *TP53* mutation harboured higher intra-tumour heterogeneity than those without the *TP53* mutation [**Figure S10**, **Table S8**]. Somatic mutational signatures associated with spontaneous deamination of cytosine, tobacco, APOBEC-activity, and defective DNA repair, were found in a substantial proportion of female patients; these were also found to be high in previous studies on oral[1,11,13]. Our finding of aflatoxin (caused primarily by *Aspergillus flavus* and *Aspergillus parasiticus* and found in crops) driven somatic mutations[21] in a female patients is novel. In this cohort of female OSCC patients, we found a significant enrichment of somatic alterations in -(a) RNA Polymerase II transcription, (b) Notch signalling, (c) PI3K-Akt signalling, (d) Integrin signalling, (e) Calcium signalling, (f) cAMP signalling, (g) Wnt signalling, and (h) Cadherin signalling pathways. These altered pathways can be targeted to alter disease prognosis. In our previous study[13] it was observed that *NOTCH1* deletion was an early feature in tumourigenesis. Mutation-based inactivation of several WNT pathway members have been identified as a major mechanism underlying tumour pathogenicity in multiple cancer types (notably colorectal, stomach and bladder) in the TCGA cohort[52,53]; our observation in oral cancer is consistent with the earlier findings. In addition, somatic mutations in integrin and cadherin signalling pathway members were observed, which are known to regulate normal cell-cell interaction and integrity of extracellular matrix, hindering cell migration and invasion[54]. FDA-approved drugs, alpelisib and capivasertib against *PIK3CA* and tipifarnib against *HRAS* oncogenic mutations are actively used in treatment of various other

cancer types such as breast and cervical[55,56]; about 29% of the patients in our female oral cancer cohort acquired one of these mutations and hence may benefit if these drugs are repurposed. In 8 patients (21%, n=38) of our cohort, EGFR amplification occurred without a concomitant *EPHA2* somatic mutation - indicating co-occurrence of functional EPHA2 with EGFR amplification. This finding agrees with previous studies that showed activated EGFR-Functional EPHA2 interaction promote tumorigenesis in lung and colorectal cancers[57]. Cetuximab is already in clinical use against *EGFR* in oral cancer[58] which is amplified in about 22% patients in this cohort. Integration of histopathology (H&E) 20X whole slide images (WSIs) with the genome alteration data for a subset of patients, revealed that - (a) in *EGFR* amplified tumours there was very low T-leukocyte infiltration and (b) tumours with oncogenic mutations in *TP53*, *PIK3CA* or *HRAS* had high T-leukocyte infiltration; these observations were supported by immune deconvolution analysis on TCGA-HNSCC data. This indicates, *EGFR* amplified tumours harbouring immunodepleted microenvironment may not be good candidate for immunotherapy; rather immunotherapy may be applicable to patients with oncogenic *TP53, PIK3CA* or *HRAS* mutations. In non-small cell lung cancer (NSCLC), dual PD-1/VEGFR2 blockade have been employed to salvage CD8+ T cell response after EGFR inhibition[59,60]. Similar therapeutic options may be used in Indian oral cancer patients for countering immunosuppressive tumour microenvironment and consequent tumour aggressiveness. We also observed that the incidence of lymph node metastasis is over 2-fold (odds ratio = 2.5) higher among patients with low immune infiltration, consistent with findings of earlier studies that these aggressive tumours often modify the immune milieu to be more favourable for their growth and survival[61,62].

We provide deeper elucidation of the immune-depleted nature of EGFR-positive head and neck tumors through analysing large single-cell RNA sequencing datasets. Anti-tumorigenic CD8+ T, CD4+ T, as well as NK cells were found to be depleted in EGFR-High tumours. We identified that EGFR-High malignant cells actively evade immune recognition by several mechanisms, which includes: 1) downregulation of antigen-presentation via MHC-molecules (both HLA-class I and II, and B2M), 2) overexpression of several pro-angiogenic (VEGFB), and 3) overexpression of glutathione metabolism-related genes (GPX2, AKR1C1, etc.). Impaired glutathione metabolism has been linked to facilitate Treg-like state and overall immune suppression[25,63]. We also identified several epithelial-immune interactions by which EGFR-High malignant cells actively drive the immune-suppressed state of the TME. We identified that EGFR-High malignant cells influence the local immune milieu by expression of ligands like PTN, MDK, PLAU. EGFR-High tumours had enriched CCL20-CCR6 interactions

between epithelial and immune cells, which has been known to promote a Treg-like state[64]. Expression of ligands, like LGALS3 by EGFR-High malignant cells leads to an exhausted and tolerogenic CD8+ T cell population in EGFR-High tumours by induction of LAG3, CD69 and CD44. Using integrative analysis of genomic and single-cell transcriptomic data, we have identified two actionable groups of patients based on their distinct molecular features - (1) characterised by expression of LAG3, PTN, MDK who may benefit from immunotherapeutic modalities, and (2) characterised by PIK3CA/HRAS oncogenic mutations who may benefit from targeted therapy.

Our study is a unique contribution to somatic genomics of oral tumours among female patients, who have been grossly underrepresented in previous studies. Our study has revealed several novel features of genomic alteration among these female patients. We have also identified some clinically actionable targets in some molecular subgroups of patients.

References

1. India Project Team of the International Cancer Genome Consortium. Mutational landscape of gingivo-buccal oral squamous cell carcinoma reveals new recurrently-mutated genes and molecular subgroups. *Nat Commun* 4, 2873 (2013).
2. Upadhyay, P. *et al.* Genomic characterization of tobacco/nut chewing HPV-negative early stage tongue tumors identify MMP10 asa candidate to predict metastases. *Oral Oncol* 73, 56–64 (2017).
3. Mathur, P. *et al.* Cancer Statistics, 2020: Report From National Cancer Registry Programme, India. *JCO Glob Oncol* 1063–1075 (2020) doi:10.1200/GO.20.00122.
4. Özdemir, B. C. & Dotto, G.-P. Racial Differences in Cancer Susceptibility and Survival: More Than the Color of the Skin? *Trends Cancer* 3, 181–197 (2017).
5. Rubin, J. B. The spectrum of sex differences in cancer. *Trends Cancer* 8, 303–315 (2022).
6. Zhou, Y. *et al.* P53 codon 72 polymorphism and gastric cancer: A meta-analysis of the literature. *Int J Cancer* 121, 1481–1486 (2007).
7. Katkoori, V. R. *et al.* Prognostic Significance of p53 Codon 72 Polymorphism Differs with Race in Colorectal Adenocarcinoma. *Clinical Cancer Research* 15, 2406–2416 (2009).
8. Cardano, M., Buscemi, G. & Zannini, L. Sex disparities in DNA damage response pathways: Novel determinants in cancer formation and therapy. *iScience* 25, 103875 (2022).
9. Klein, S. L. & Flanagan, K. L. Sex differences in immune responses. *Nat Rev Immunol* 16, 626–638 (2016).
10. Rubin, J. B. *et al.* Sex differences in cancer mechanisms. *Biol Sex Differ* 11, 17 (2020).
11. Lawrence, M. S. *et al.* Comprehensive genomic characterization of head and neck squamous cell carcinomas. *Nature* 517, 576–582 (2015).
12. Massey, S. C. *et al.* Sex differences in health and disease: A review of biological sex differences relevant to cancer with a spotlight on glioma. *Cancer Lett* 498, 178–187 (2021).
13. Ghosh, A. *et al.* Integrative analysis of genomic and transcriptomic data of normal, tumour and co-occurring leukoplakia tissue triads drawn from patients with gingivobuccal oral cancer identifies signatures of tumour initiation and progression. *J Pathol* (2022) doi:10.1002/path.5900.
14. Bailey, M. H. *et al.* Comprehensive Characterization of Cancer Driver Genes and Mutations. *Cell* 173, 371-385.e18 (2018).
15. Pradhan, S. *et al.* dbGENVOC: database of GENomic Variants of Oral Cancer, with special reference to India. *Database* 2021, baab034 (2021).
16. Rasti, A. R. *et al.* PIK3CA Mutations Drive Therapeutic Resistance in Human Epidermal Growth Factor Receptor 2–Positive Breast Cancer. *JCO Precis Oncol* e2100370 (2022) doi:10.1200/PO.21.00370.
17. Prior, I. A., Lewis, P. D. & Mattos, C. A Comprehensive Survey of Ras Mutations in Cancer. *Cancer Res* 72, 2457–2467 (2012).
18. Glover, T. W., Wilson, T. E. & Arlt, M. F. Fragile sites in cancer: more than meets the eye. *Nat Rev Cancer* 17, 489–501 (2017).

19. Degasperi, A. *et al.* A practical framework and online tool for mutational signature analyses show intertissue variation and driver dependencies. *Nat Cancer* 1, 249–263 (2020).
20. Alexandrov, L. B. *et al.* The repertoire of mutational signatures in human cancer. *Nature* 578, 94–101 (2020).
21. McCullough, A. K. & Lloyd, R. S. Mechanisms underlying aflatoxin-associated mutagenesis – Implications in carcinogenesis. *DNA Repair (Amst)* 77, 76–86 (2019).
22. Faden, D. L. *et al.* APOBEC mutagenesis is tightly linked to the immune landscape and immunotherapy biomarkers in head and neck squamous cell carcinoma. *Oral Oncol* 96, 140–147 (2019).
23. Xiao, G. *et al.* Heterogeneity of tumor immune microenvironment of EGFR/ALK-positive tumors versus EGFR/ALK-negative tumors in resected brain metastases from lung adenocarcinoma. *J Immunother Cancer* 11, e006243 (2023).
24. Maron, S. B. *et al.* Targeted Therapies for Targeted Populations: Anti-EGFR Treatment for EGFR-Amplified Gastroesophageal Adenocarcinoma. *Cancer Discov* 8, 696–713 (2018).
25. Ahmed, K. M. *et al.* Glutathione peroxidase 2 is a metabolic driver of the tumor immune microenvironment and immune checkpoint inhibitor response. *J Immunother Cancer* 10, e004752 (2022).
26. Chen, P. *et al.* Tumor-Associated Macrophages Promote Angiogenesis and Melanoma Growth via Adrenomedullin in a Paracrine and Autocrine Manner. *Clinical Cancer Research* 17, 7230–7239 (2011).
27. Larkin, J. *et al.* CD44 Differentially Activates Mouse NK T Cells and Conventional T Cells1. *The Journal of Immunology* 177, 268–279 (2006).
28. Kong, T. *et al.* CD44 Promotes PD-L1 Expression and Its Tumor-Intrinsic Function in Breast and Lung Cancers. *Cancer Res* 80, 444–457 (2020).
29. Hou, W., Kong, L., Hou, Z. & Ji, H. CD44 is a prognostic biomarker and correlated with immune infiltrates in gastric cancer. *BMC Med Genomics* 15, 225 (2022).
30. Chang, Y. *et al.* Secretion of pleiotrophin stimulates breast cancer progression through remodeling of the tumor microenvironment. *Proceedings of the National Academy of Sciences* 104, 10888–10893 (2007).
31. Ubil, E. *et al.* Tumor-secreted Pros1 inhibits macrophage M1 polarization to reduce antitumor immune response. *J Clin Invest* 128, 2356–2369 (2018).
32. Pribila, J. T., Quale, A. C., Mueller, K. L. & Shimizu, Y. Integrins and T Cell–Mediated Immunity. *Annu Rev Immunol* 22, 157–180 (2004).
33. Klement, J. D. *et al.* An osteopontin/CD44 immune checkpoint controls CD8+ T cell activation and tumor immune evasion. *J Clin Invest* 128, 5549–5560 (2018).
34. Shetty, P. *et al.* Cell surface interaction of annexin A2 and galectin-3 modulates epidermal growth factor receptor signaling in Her-2 negative breast cancer cells. *Mol Cell Biochem* 411, 221–233 (2016).
35. Colomb, F. *et al.* Galectin-3 interacts with the cell-surface glycoprotein CD146 (MCAM, MUC18) and induces secretion of metastasis-promoting cytokines from vascular endothelial cells. *Journal of Biological Chemistry* 292, 8381–8389 (2017).
36. Cibrián, D. & Sánchez-Madrid, F. CD69: from activation marker to metabolic gatekeeper. *Eur J Immunol* 47, 946–953 (2017).
37. Li, Q., Tie, Y., Alu, A., Ma, X. & Shi, H. Targeted therapy for head and neck cancer: signaling pathways and clinical studies. *Signal Transduct Target Ther* 8, 31 (2023).

38. Thudium, K. *et al.* Preclinical Characterization of Relatlimab, a Human LAG-3–Blocking Antibody, Alone or in Combination with Nivolumab. *Cancer Immunol Res* 10, 1175–1189 (2022).
39. Ascierto, P. A. *et al.* Nivolumab and Relatlimab in Patients With Advanced Melanoma That Had Progressed on Anti–Programmed Death-1/Programmed Death Ligand 1 Therapy: Results From the Phase I/IIa RELATIVITY-020 Trial. *Journal of Clinical Oncology* 41, 2724–2735 (2023).
40. Heinemann, V. *et al.* Phase II randomised proof-of-concept study of the urokinase inhibitor upamostat (WX-671) in combination with gemcitabine compared with gemcitabine alone in patients with non-resectable, locally advanced pancreatic cancer. *Br J Cancer* 108, 766–770 (2013).
41. H, P. C. *et al.* A Randomized, Placebo-Controlled Trial of Natalizumab for Relapsing Multiple Sclerosis. *New England Journal of Medicine* 354, 899–910 (2025).
42. Takeuchi, H. Midkine and multiple sclerosis. *Br J Pharmacol* 171, 931–935 (2014).
43. Fabrice, A. *et al.* Alpelisib for PIK3CA-Mutated, Hormone Receptor–Positive Advanced Breast Cancer. *New England Journal of Medicine* 380, 1929–1940 (2019).
44. Hanna, G. J. *et al.* A phase 1/2 trial to evaluate the safety and antitumor activity of tipifarnib and alpelisib for patients with PIK3CA-mutated/amplified and/or HRAS-overexpressing recurrent/metastatic head and neck squamous cell carcinoma. *Journal of Clinical Oncology* 40, TPS6104–TPS6104 (2025).
45. Ho, A. L. *et al.* Tipifarnib in Head and Neck Squamous Cell Carcinoma With HRAS Mutations. *Journal of Clinical Oncology* 39, 1856–1864 (2021).
46. Zhao, M. *et al.* T cell dynamics with neoadjuvant immunotherapy in head and neck cancer. *Nat Rev Clin Oncol* 22, 83–94 (2025).
47. Biswas, N. K. *et al.* Lymph node metastasis in oral cancer is strongly associated with chromosomal instability and DNA repair defects. *Int J Cancer* 145, 2568–2579 (2019).
48. Mantovani, F., Collavin, L. & Del Sal, G. Mutant p53 as a guardian of the cancer cell. *Cell Death Differ* 26, 199–212 (2019).
49. Baugh, E. H., Ke, H., Levine, A. J., Bonneau, R. A. & Chan, C. S. Why are there hotspot mutations in the TP53 gene in human cancers? *Cell Death Differ* 25, 154–160 (2018).
50. Singh, S. *et al.* The cancer-associated, gain-of-function TP53 variant P152Lp53 activates multiple signaling pathways implicated in tumorigenesis. *Journal of Biological Chemistry* 294, 14081–14095 (2019).
51. Pasquale, E. B. Eph receptors and ephrins in cancer progression. *Nat Rev Cancer* 24, 5–27 (2024).
52. Bugter, J. M., Fenderico, N. & Maurice, M. M. Mutations and mechanisms of WNT pathway tumour suppressors in cancer. *Nat Rev Cancer* 21, 5–21 (2021).
53. Cerami, E. *et al.* The cBio Cancer Genomics Portal: An Open Platform for Exploring Multidimensional Cancer Genomics Data. *Cancer Discov* 2, 401–404 (2012).
54. Hamidi, H. & Ivaska, J. Every step of the way: integrins in cancer progression and metastasis. *Nat Rev Cancer* 18, 533–548 (2018).
55. Suehnholz, S. P. *et al.* Quantifying the Expanding Landscape of Clinical Actionability for Patients with Cancer. *Cancer Discov* 14, 49–65 (2024).
56. Chakravarty, D. *et al.* OncoKB: A Precision Oncology Knowledge Base. *JCO Precis Oncol* 1–16 (2017) doi:10.1200/PO.17.00011.
57. Kim, J., Chang, I.-Y. & You, H. J. Interactions between EGFR and EphA2 promote tumorigenesis through the action of Ephexin1. *Cell Death Dis* 13, 528 (2022).

58. Noronha Vijay M.; Joshi Amit; Bhattacharjee Atanu; Paul Davinder; Dhumal Sachin; Juvekar Shashikant; Arya Supreeta; Prabhash Kumar, V. P. A tertiary care experience with paclitaxel and cetuximab as palliative chemotherapy in platinum sensitive and nonsensitive in head and neck cancers. *South Asian J Cancer* 06, 11–14 (2017).
59. Nishii, K. *et al.* CD8+ T-cell Responses Are Boosted by Dual PD-1/VEGFR2 Blockade after EGFR Inhibition in Egfr-Mutant Lung Cancer. *Cancer Immunol Res* 10, 1111–1126 (2022).
60. Eschweiler, S. *et al.* JAML immunotherapy targets recently activated tumor-infiltrating CD8+ T cells. *Cell Rep* 42, 112040 (2023).
61. Reticker-Flynn, N. E. *et al.* Lymph node colonization induces tumor-immune tolerance to promote distant metastasis. *Cell* 185, 1924-1942.e23 (2022).
62. Peng, J.-M. & Su, Y.-L. Lymph node metastasis and tumor-educated immune tolerance: Potential therapeutic targets against distant metastasis. *Biochem Pharmacol* 215, 115731 (2023).
63. Xu, C. *et al.* The glutathione peroxidase Gpx4 prevents lipid peroxidation and ferroptosis to sustain Treg cell activation and suppression of antitumor immunity. *Cell Rep* 35, (2021).
64. Lian, J. *et al.* Eomes promotes esophageal carcinoma progression by recruiting Treg cells through the CCL20-CCR6 pathway. *Cancer Sci* 112, 144–154 (2021).
